# ﻿Ionizing radiation resilience: how metabolically active lichens endure exposure to the simulated Mars atmosphere

**DOI:** 10.3897/imafungus.16.145477

**Published:** 2025-03-31

**Authors:** Kaja Skubała, Karolina Chowaniec, Mirosław Kowaliński, Tomasz Mrozek, Jarosław Bąkała, Ewa Latkowska, Beata Myśliwa-Kurdziel

**Affiliations:** 1 Institute of Botany, Faculty of Biology, Jagiellonian University, Gronostajowa 3, 30-387 Kraków, Poland; 2 Doctoral School of Exact and Natural Sciences, Jagiellonian University in Kraków, Prof. S. Łojasiewicza 11, 30-348, Kraków, Poland; 3 Space Research Centre, Polish Academy of Sciences, Bartycka 18a, 00-716 Warsaw, Poland; 4 Laboratory of Metabolomics, Faculty of Biochemistry, Biophysics and Biotechnology, Jagiellonian University, Gronostajowa 7, 30-387, Kraków, Poland; 5 Department of Plant Physiology and Biochemistry, Faculty of Biochemistry, Biophysics and Biotechnology, Jagiellonian University, Gronostajowa 7, 30-387 Kraków, Poland

**Keywords:** Adaptation, *Cetrariaaculeata*, *Diploschistesmuscorum*, extremotolerance, habitability, lichen, Mars conditions, X-rays

## Abstract

To deepen our understanding of lichen adaptation and their potential to colonize extraterrestrial environments, we aimed to identify physiological/biochemical responses of selected lichen species in a metabolically active state to simulated Mars-like conditions in the dark including exposure to X-rays. Our study is the first to demonstrate that the metabolism of the fungal partner in lichen symbiosis was active while being in a Mars-like environment. *Diploschistesmuscorum* was able to activate defense mechanisms effectively. In contrast, increased oxidative stress and associated damage were not effectively balanced in *C.aculeata*, which does not support the melanin’s radioprotective function in this species. The heavy crystalline deposit on *D.muscorum* thallus might offer protection enhancing lichen resistance to extreme conditions. We concluded that metabolically active *D.muscorum* can withstand the X-ray dose expected on the Mars surface over one year of strong solar activity. Consequently, X-rays associated with solar flares and SEPs reaching Mars should not affect the potential habitability of lichens on this planet.

## ﻿Introduction

Lichens inhabit diverse ecosystems worldwide, but they are particularly crucial in extreme environments like hot deserts and cold Polar regions. They are known as extremophiles, able to survive under extreme temperatures, intense radiation, and prolonged water scarcity (Rothschild and Mancinelli 2001; Armstrong 2019). The remarkable ability of lichens to endure harsh conditions has led to the suggestion that they are well-suited to survive the extreme environment of outer space (Sancho et al. 2008). Lichen successful life strategy depends on the symbiotic association between a fungus and an alga or cyanobacteria, which allows them to colonize extreme terrestrial habitats where no other multicellular organism is able to survive (de la Torre Noetzel and Sancho 2020). The key to understanding their impressive resistance lies in their characteristics of ‘stress-tolerant’ organisms, i.e., low metabolic rates, minimal nutritional requirements, and extended longevity, which are further supported by protective mechanisms: radiation screening, thermal dissipation, and antioxidant defense (Kranner et al. 2005; Nguyen et al. 2013). Moreover, they can cope with prolonged water scarcity or even a total absence of liquid water (Lange et al. 1986). This is related to the lack of ability to regulate water content (poikilohydry), which allows them to survive long periods of severe desiccation without damage in the dormant state (Honegger 2007), but also to tolerate high UV/PAR levels and temperature extremes associated with drought conditions (Nybakken et al. 2004). This involves a transition into an ametabolic state known as anhydrobiosis (Kranner et al. 2005). Notably, upon rehydration lichen metabolism is reactivated (Kranner et al. 2008). Next adaptive traits concern specific thallus structure, a diverse set of morphological/anatomical properties, and high phenotypic plasticity providing adaptation to local microclimatic conditions (Osyczka and Rola 2013). So far, several attributes that enable lichens to survive in extreme conditions have been identified including the structure of the cortex (de Vera et al. 2003), algal arrangement and presence of calcium oxalate crystals (Meeßen et al. 2013), non-reducing sugars maintaining membrane structure (Crowe et al. 1998), enzymatic and non-enzymatic antioxidants (Gasulla et al. 2021) and UV-screening secondary metabolites and melanin pigments (Solhaug et al. 2003; Mafole et al. 2019). These adaptations may have potential implications for their ability to colonize extraterrestrial environments.

Mars is a primary focus of interest in astrobiology due to the presence of water and the associated potential for life. The present atmospheric conditions on Mars are inhospitable and thus the potential habitats for existing life are limited (Jakosky 2024). Nevertheless, habitable environments may exist below or on the surface during more favorable climatic periods (McCollom 2006). These niches could act as isolated habitats that protect from harsh conditions (de Vera et al. 2014). Despite the atmosphere being mostly composed of carbon dioxide (~95%), the effectiveness of greenhouse warming is limited (Trainer et al. 2019). The temperature on Mars predominantly remains below the freezing point of water and the atmospheric pressure is c.a. 6 millibars (Nakagawa 2019). Consequently, a considerable part of the existing water on Mars is ice and atmospheric water vapor; however, certain water amounts could be temporarily present as liquid water (Nazari-Sharabian et al. 2020). Both ionizing and non-ionizing radiation constantly reach the Mars surface and pass through the atmosphere of Mars much more easily than on Earth (Guo et al. 2021). Since UV and ionizing radiation are extremely harmful to living organisms, this factor is the most limiting in the context of habitability on Mars (Checinska Sielaff and Smith 2019).

Several experiments tested the response of lichens after exposure to Mars-like conditions on board space missions or ground-based simulated Mars conditions and included factors such as atmospheric pressure and gas composition, temperature, and UV radiation (e.g., de Vera et al. 2010; Sánchez et al. 2012; Brandt et al. 2015; de la Torre Noetzel et al. 2018; Lorenz et al. 2023). The conducted experiments were short (from 5 days) or long-term (up to 559 days); however, the results were generally similar and demonstrated the high resistance of lichens in the anhydrobiotic state. Most studies focused on assessing cell viability using LIVE/DEAD staining, and the results indicated high viability rates for both photobiont and mycobiont cells (de Vera et al. 2010; Brandt et al. 2015). The second commonly studied parameter was chlorophyll *a* fluorescence, an indicator of the active light reaction of photosynthesis, and the vast majority of studies showed recovery of the PSII activity or even revealed unaltered photosynthetic performance (e.g., Sánchez et al. 2012). The aforementioned experiments focused on the survival and regeneration of lichens after exposure. A groundbreaking work demonstrated that lichen *Pleopsidiumchlorophanum* can continue photosynthesis under Mars-like conditions in protected niche conditions for 34 days (de Vera et al. 2014). A subsequent study confirmed this ability for *Xanthoriaparietina* (Lorenz et al. 2023). In contrast, *Circinariagyrosa* failed to perform photosynthesis under simulated Mars-like conditions (de la Torre Noetzel et al. 2018). While photobiont survival is crucial for lichen symbiosis, to date very little is known about the physiological condition of the mycobiont. Previous studies did not find DNA damage in fungal components but revealed changes in the levels of certain sterols and demonstrated overproduction of antioxidants following exposure to Mars-like conditions (de la Torre Noetzel et al. 2018; Lorenz et al. 2024). Finally, none of these experiments considered the impact of ionizing radiation occurring on Mars’s surface.

Ionizing radiation is the most challenging factor for the possibility of life beyond the Earth (Nelson 2003). It induces water radiolysis that triggers cellular damage by reactive oxygen species (ROS) and associated oxidative damage (De Micco et al. 2011). The exposure to ionizing radiation may also induce disturbances in genetic, morphological, physiological, and biochemical processes, which differ depending on the species, radiation dose, and radiation type (Gudkov et al. 2019). So far, the majority of astrobiological studies concentrated on the effect of UV radiation on lichens, whereas the effect of exposure to ionizing radiation was the subject of only a few studies (Brandt et al. 2017; de la Torre Noetzel et al. 2017). Cosmic ionizing radiation on Mars comes from X-rays and energetic particles, including galactic cosmic rays (GCR) and solar energetic particles (SEP) (Semkova et al. 2023). In situ measurements on the Mars surface showed an average dose rate of 0.21+/-0.04 mGy/day related to GCR (Hassler et al. 2014). SEPs are highly variable and may provide doses several orders of magnitude larger than GCR in time scales of days (Hassler et al. 2014). The latter is related to solar X-ray emission that may change in minutes to hours’ timescales in the case of solar flares, or years due to the 11-year solar cycle (Smith and Scalo 2008; Semkova et al. 2023), resulting in the potential for high dose rates. Apart from single-strong events, the Sun may experience enhanced activity, when the background X-ray radiation exceeds 102–103 times the quiet period resulting in much more radiation doses than in the case of the strongest flare. Since large doses of X-rays periodically reach the surface of Mars, understanding their impact on lichen survival is crucial.

Although several model lichen species have been used in astrobiological studies, many questions and knowledge gaps remain, and addressing them will deepen our understanding of lichen adaptation and their potential to colonize extraterrestrial environments. First, most previous studies focused mainly on cell viability and photosynthetic efficiency, while knowledge about physiological/biochemical changes and potential adaptations to these conditions is still insufficient. The second large knowledge gap concerns the lack of detailed studies on the physiological condition of the mycobiont since the vast majority of studies focused on the photobiont. Because lichens are obligate symbionts, this aspect plays a key role in the potential of life to survive and reproduce in extraterrestrial environments. Third, most studies concerned resistance limits of lichens in the anhydrobiotic state, in which their ability to survive extremes without damage has been proven. Although this is of great importance in the context of potential interplanetary transfer, as regards colonization of extraterrestrial habitats and successful establishment of lichen symbiosis, more information concerning lichens in the metabolically active state is needed. Previous experiments on the effect of Mars-like conditions on lichens focused solely on reproducing atmosphere composition, temperature, pressure, humidity, and solar radiation, whereas ionizing radiation has not been taken into account. Finally, our knowledge on the interaction of radiation with cellular water molecules in a process called radiolysis in hydrated lichens has not been deepened. To expand our understanding of these aspects, we set two main objectives. First, we wanted to identify the responses of two selected lichen species placed in a metabolically active state in a simulation facility reproducing Mars-like conditions in the dark including X radiation, to determine their effects at various levels of the structure and functioning of the thallus. Moreover, we aimed to answer key questions regarding lichen traits that provide them greater stress resistance. Therefore, we selected two lichen species differing in growth form, thallus anatomical organization, and the presence of specific traits that could potentially serve as adaptations to excessive X-ray exposure. Consequently, we aimed to identify the effectiveness of protective mechanisms enabling the survival of these lichens by determining the mutual relations between the degree of damage and triggering a response to this damage by analyzing various functional traits.

## ﻿Materials and methods

### ﻿Target lichen species

Because lichen morphology/anatomy and biochemistry are key factors in understanding the extreme tolerance of lichens, we selected for study two species with different potential adaptation traits to extraterrestrial conditions, particularly ionizing radiation.

*Diploschistesmuscorum* (Scop.) R. Sant. is a crustose terricolous lichen occurring in dry and sunny habitats. It is known as “crater lichen” due to apothecia that are semi-immersed in the thallus. It is capable of withstanding exceptionally high concentrations of toxic trace elements (Osyczka and Rola 2019) and is considered a hyperaccumulator of Zn and Pb (Rola et al. 2019). It can increase the production of sugar alcohols under heavy-metal stress (Osyczka et al. 2021), which may turn out to be important in the context of this study since polyol production is a key mechanism involved in desiccation tolerance and antioxidant protection (Grimm et al. 2021). The production of secondary metabolites may also compensate for stress since both lecanoric and orsellinic acids produced by *D.muscorum* demonstrate high antioxidant activity (Lopes et al. 2008).

*Cetrariaaculeata* (Schreber) Fr. is a fruticose epigeic lichen with coarsely dichotomously branched thalli, forming irregular, shrubby tufts of brown or almost black color. It occurs most frequently in open polar and boreal environments from the maritime Antarctic to the high Arctic and in high mountains in wind-exposed situations (Nimis and Martellos 2024). This species has a three-layered thick cortex layer, with a dense, brown outer layer with the accumulation of dead cells (Pérez-Ortega et al. 2012) that provide a good protective barrier against excessive solar radiation. Furthermore, our previous study confirmed that the dark color of the thallus results from the presence of melanins in this species (Chowaniec et al. 2024). Melanin pigments in lichens act as a sunscreen, especially reducing UVB and UVA wavelengths (Mafole et al. 2019), and are also powerful antioxidants (Jacobson et al. 1995), which can contribute to the reduction of oxidative stress. Melanization is common in lichens growing in harsh environments and constitutes an effective protection of lichen photobionts (Nguyen et al. 2013). Moreover, the resistance of melanized free-living fungi to cosmic and terrestrial ionizing radiation suggests that melanin also plays a pivotal role in radioprotection (Pacelli et al. 2017).

### ﻿Experiment equipment

**Figure 1.**
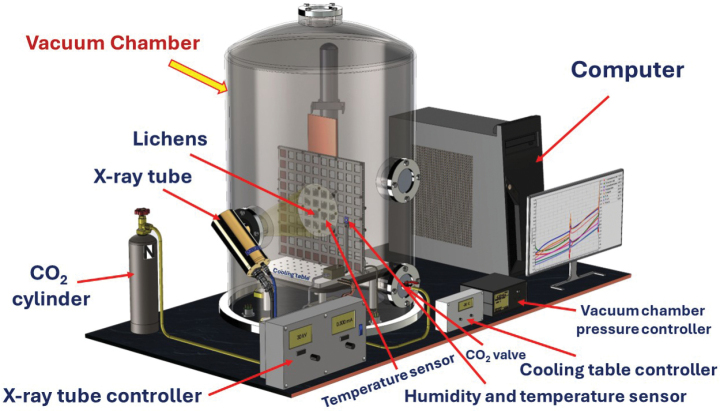
Experiment arrangement of vacuum chamber with the additional facility, including metal grate with lichens, cooling table, temperature, pressure and humidity sensors, X-ray lamp with the controller, CO_2_ valve with cylinder, controllers of vacuum chamber, pressure, cooling table, and computer.

The experiments were performed at the Space Research Centre of the Polish Academy of Sciences (CBK PAN) laboratory. The facility was specially constructed for this project. The main part of the facility was a vacuum chamber that constitutes a stainless-steel cylinder with inside dimensions of 60 cm height, and 52 cm width (volume of ~127 L). The chamber was equipped with flanges to which an X-ray tube and a CO_2_ supply hose were connected (Fig. 1). The X-ray source was Jupiter 5000 Series Radiation Shielded X-ray Tube (Oxford Instruments). The cooling table connected to the cooling unit was installed inside the chamber. Temperature and humidity were monitored during the experiments by sensors placed on the grate with lichen samples and connected to the computer, while external controllers regulated the X-ray tube. The X-ray tube was operated at a voltage of 30 kV and a current of 0.5 mA. The inside pressure was controlled by a vacuum pump (Unitra DZ2000, UNITRA-UNIMA Zakład Techniki Próżniowej w Koszalinie, Poland). The lichen samples were attached to the metal base mounted exactly perpendicular to the X-ray source at a distance of 35 cm (Suppl. material 1: fig. S1).

### ﻿Experimental conditions

Since UV radiation – the best studied and at the same time one of the most detrimental factors associated with exposure to the surface of Mars has already been thoroughly studied in the context of lichens, in our experiment we decided to exclude this factor and focus on the Mars-like conditions including the factor of ionizing radiation, which was not included in previous experiments. To the best of our knowledge, such an experimental design has not been applied to lichens in a metabolically active state so far. Lichen thalli were hydrated by spraying with water, and metabolically active lichens were placed on a metal grid inside the chamber. The conditions inside the chamber were set to simulate Mars-like conditions. The experiments were conducted separately for each species and lasted 5 hours. The first two-and-a-half hours simulated the temperature on the surface of Mars during the day to enable metabolic processes to occur. Then the temperature was gradually lowered to simulate the temperature drop that occurs on the surface of Mars at night. During the experiment, the lichens received an X-ray radiation dose of 50 Gy, which is possible to reach the surface of Mars during one year of strong solar activity (see section Estimation of X-ray doses at Mars surface). Inside the chamber, the pressure was kept within the range of 5–7 mbar and the gas composition in the chamber during the experiment was set to 95% CO_2_ and 5% air (see Suppl. material 1: table S3). The temperature varied between 18°C during the day simulation and -26°C during the night simulation, while relative humidity right next to the lichen samples ranged from 8–32%. Experimental conditions are summarized in Suppl. material 1: table S3. The experiment planned in this way allowed us to freeze the lichen samples and thus inhibit their metabolism at the end of the experiment. Immediately after the experiment, the samples were transferred to a portable freezer at -25°C and transported to the laboratory, where they were placed in a freezer at -30°C until the physiological and biochemical analyses were performed. The temperature was not allowed to rise during this time, to prevent the start of any metabolic processes. Thanks to this, the results of the analyses carried out reflect the physiological state of the lichen thalli during the experiment. During exposure, we were unable to measure the desiccation kinetics of the thalli under experimental conditions to confirm metabolic activity during the experiment. Nevertheless, after the end of the experiment, we selected a small amount of lichen material to measure thallus water content (WC) by the impedance technique (Schuster et al. 2002). The readings were in the range of 14–16%, which indicates that lichen thalli were not completely dried. Consequently, it can be assumed that during a certain part of the experiment, at least minimal metabolic activity of the fungal and algal cells was maintained.

At the same time in parallel to the experiment, control samples were hydrated and placed on flat trays in a climatic chamber in darkness (KK 115 FIT D Smart PRO, Pol-Eko, Poland) for 5 h (relative humidity 90%, temperature 15°C), then frozen at -30°C until physiological and biochemical analyses.

### ﻿Estimation of X-ray doses at Mars surface

Cumulative radiation dose on the Martian surface is difficult for a precise determination as it contains highly unpredictable events like solar flares and SEPs whose fluence covers several orders of magnitude and whose occurrence is still unpredictable. The most reliable estimate is GCR as it fluctuates within several percent and is measured routinely at Mars’s surface by various experiments. Using data from the Curiosity rover, Hassler et al. (2014) reported a mean value of GCR equal to 0.21+/-0.04 µGy/day measured in the 300-day long period around solar maximum. As GCR intensity is anti-correlated to the solar cycle (Ross and Chaplin 2019), we may treat this value as minimal. Accumulating it over one year and an entire solar cycle we obtain radiation doses from 0.08 to 1 Gy. The second ingredient is SEPs which are potentially far more dangerous for living organisms. Semkova et al. (2023) reported five SEPs detected with instruments onboard ExoMars close to the minimum phase of the solar cycle 24^th^. The strongest dose measured was 13.8+/-1.4 mGy for the SEP event observed from 15 to 19 February 2022. Hassler et al. (2014) reported one weak SEP effect measured on the Mars surface. The dose estimated was small, similar to GCR accumulated over one day. However, the strongest SEPs measured in history were several orders (10^3^–10^5^) of magnitude stronger in the peak flux and lasted significantly longer (https://cdaw.gsfc.nasa.gov/CME_list/sepe/). Assuming the SEP which is 10^5^ stronger than the one measured by Hassler et al. (2014) we estimate that a single extreme SEP event may deliver dose up to 5 Gy on the Martian surface. Such strong events may occur in series like October-November 2003, causing severe threats to living organisms. Thus, we estimate that a dose of about 20 Gy may be achieved within one year during high solar activity. In addition, strong SEPs are related to strong solar flares emitting intense X-ray radiation. Smith and Scalo (2007) estimate doses of 1–10 Gy from flares of total energy in the range 6×10^33^–6×10^34^. The strongest solar flare recorded up to date emitted 1.9 ± 0.7×10^33^ erg, however stronger (10 times) ones are possible (Cliver et al. 2022). Taking into account all these values we estimated that a total dose of 50 Gy is moderately possible during one year of strong solar activity and very reliable during one, strong solar cycle (11 years).

### ﻿Morphological and anatomical analysis

To visualize anatomical features of the studied species, cross-sections of the lichen thalli were made and observed under a Nikon Eclipse 80i light microscope. *D.muscorum* thallus was stained with a lactophenol blue solution to better visualize calcium oxalate crystals. Micromorphology of lichen thalli was examined using a scanning electron microscope (SEM). The air-dried lichen samples were observed using a HITACHI S-4700 with NORAN Vantage after coating with a thin gold layer. Other samples were also analyzed after coating with carbon for elemental identification by energy-dispersive X-ray spectroscopy (EDX) to examine crystalline deposits and particles of external origin trapped inside the thallus. Both lichen surfaces and cross-sections were analyzed.

### ﻿Membrane lipid peroxidation

The level of membrane lipid peroxidation in lichen samples was measured using the TBARS assay, based on the method of Heath and Packer (1968), modified by Politycka (1996). About 40 mg of air-dried lichen were homogenized in 1.5 ml of 0.25% thiobarbituric acid (TBA) in 10% trichloroacetic acid (TCA). The mixture was heated at 95°C for 30 min, cooled, and centrifuged at 12,000 × *g* for 15 min. Absorbance was measured at 532 nm and corrected at 600 nm (Shimadzu UV-1900i, Shimadzu Corporation, Japan), and TBARS levels were calculated using the extinction coefficient of the TBA-MDA complex. Results were expressed in nmol of TBARS per gram of lichen (DW), with eight replicates per group.

### ﻿Integrity of cell membranes

Cell membrane integrity was assessed following Paoli et al. (2011), with modifications of Osyczka and Rola (2019). Ca 100 mg of each lichen sample was placed in 50 ml of distilled water. After shaking for 1 h (Vibramax 100, Heidolph Instruments, Germany), the initial conductivity (*Ci*) was measured (Seven Go Duo SG23-FK5, Mettler Toledo, Switzerland). The conductivity after shaking (*Cv*) was then recorded, followed by boiling the samples for 10 min to destroy the membranes and measuring final conductivity (*Cf*). The relative electrical conductivity (EC), indicating membrane integrity loss, was calculated as ((*Cv* - *Ci*)/*Cf*) × 100%. Eight replicates were used per group.

### ﻿Dehydrogenase activity

The vitality of the mycobiont, about 90% of lichen biomass, was assessed by the reduction of 2,3,5-triphenyltetrazolium chloride (TTC) to red triphenylformazan (TPF), reflecting dehydrogenase activity and representing the activity of the mitochondrial respiratory chain (Bačkor and Fahselt 2005). TTC constitutes an artificial electron acceptor and receives electrons directly from the low-potential cofactors of NADH dehydrogenase (complex I) in the mitochondrial respiratory chain (Rich et al. 2001). Ca. 40 mg of lichen material was incubated in 0.6% TTC and 0.005% Triton X 100 solution in 50 mM sodium phosphate buffer for 20 h in the dark at 25°C. After rinsing and drying of samples on filter paper, formazan was extracted with 6 ml of ethanol (96%) at 65°C for 1 h and the absorbance of supernatant was measured at 485 nm. Results were expressed as absorbance per dry weight of the thalli based on eight replicates.

### ﻿Hydrogen peroxide assay

Lichen samples (ca. 50 mg DW) were homogenized in sodium phosphate buffer (pH 6.8) with the addition of polyvinylpyrrolidone and centrifuged at 10,000 × *g* for 5 min. To the 1 ml of supernatant 0.2 ml of 10% (v/w) TiCl_4_ in 96% HCl and 0.2 ml of 36% ammonia solution (v/v) was added. Next samples were centrifuged at 10,000 × *g* for 15 min and the sediment was washed with ice-cold acetone (99%) and centrifuged again. This step was repeated if necessary. In the end, 3 ml of 20% H_2_SO_4_ was added to the sample and left for 15 min at room temperature for color development and the absorbance was measured at 410 and 415 nm. The concentration of H_2_O_2_ was determined using the calibration curve. Eight replicates were used per group.

### ﻿Sugar alcohols

The determination of sugar alcohol content was carried out in a multi-step procedure previously described in detail by Osyczka et al. (2021). Briefly, sugar alcohols were extracted from powdered lichen thalli (20 mg DW) with 70% ethanol in an ultrasonic water bath (15 min, 75°C). The solvent was evaporated and the obtained residue was re-dissolved in 1 ml of deionized water. After deproteinizing the sample, the derivatization procedure of sugar alcohols was performed using benzoyl chloride. Then, after adding 500 μl of ethyl acetate to the formed benzoylated sugars, the sample was mixed, and finally, the ethyl acetate phase was taken and evaporated. The obtained residue was dissolved in 1 ml of 80% acetonitrile (v/v) and analyzed using a Nexera-i LC-2040C 3D Plus UHPLC (Shimadzu, Japan) with a PDA detector. The separation of sugar alcohols was achieved on a C18 column (ReproShell ODS-1, Dr. Maisch, Germany; 2.7 μm; 150 mm × 4.6) at 30°C using a two-solvent system: A (0.05% TFA in water; v/v) and B (0.05% TFA in acetonitrile; v/v) with linear gradient elution from 60 to 100% B for 10 min at a flow rate of 0.8 ml min^-1^. Detection was performed at 232 nm. Quantification of sugar alcohols was done based on calibration curves for D-ribitol (5–50 μg ml^-1^), D-arabitol and D-mannitol (20–200 μg ml^-1^) (Merck, USA). Eight replicates were used per group.

### ﻿Secondary metabolites

Quantitative determination of secondary metabolites of *D.muscorum* was performed with the use of UHPLC-PDA analysis according to the procedure described earlier by Osyczka et al. (2021). The identity of lichen compounds was confirmed by the UPLC–ESI–QQQ–MS/MS method. The quantity of compounds was expressed as relative amounts represented by peak areas calculated based on eight replicates.

### ﻿Glutathione determination

Both forms of glutathione, i.e., reduced (GSH) and oxidized (GSSG), were extracted by incubating ca. 70 mg of powdered lichen thalli in 600 μl of a 5% (w/v) 5-sulfosalicylic acid (SSA, Sigma-Aldrich, USA) solution, containing 6.3 mM diethylenetriaminepentaacetic acid (DTPA, Sigma-Aldrich, USA) for 10 min in an ice bath according to the procedure described by De Knecht et al. (1994). After centrifuging (15,000 × *g*, 10 min), the supernatant was divided into 2 parts: in the first part GSH was determined; in the second part, the disulfide groups (-S-S-) were reduced by dithiothreitol (DTT), and then total glutathione (sum of GSH + GSSG) was determined. The pre-column derivatization of thiol groups before HPLC analysis was performed according to Garcia et al. (2008). First, 500 μl of 0.5 M Tris-HCl buffer (Sigma-Aldrich, USA) at pH 8.9 was added to 200 μl of the extract, and then 20 μl of water (for GSH determination) or 20 μl of dithiotreitol (DTT, Merck, USA) (for determination of GSH + GSSG) was added. After 5 min of incubation, 350 μl of 10 mM Ellman’s reagent (5,5′ dithio (2-nitrobenzoic acid) – DTNB, Sigma-Aldrich, USA) in 0.5 M K_2_HPO_4_ (POCh, Poland) at pH of 8.0 was added. The sample was mixed and incubated for 5 min, then acidified with 100 μl of 7 M H_3_PO_4_ (POCh, Poland) and centrifuged (15,000 × *g*; 10 min). All steps were performed on ice. Derivatized GSH was separated on a C18 ReproShell ODS-1 column (Dr. Maisch, Germany; 2.7 μm; 150 mm × 4.6) in a Shimadzu Nexera-*i* LC-2040C 3D Plus UHPLC (Shimadzu, Japan) with a PDA detector. A detailed description of HPLC separation conditions can be found in Rola et al. 2022. GSH was detected at 330 nm (Garcia et al. 2008) and quantified based on a calibration curve prepared with the commercial standard of GSH (Sigma-Aldrich, USA) (1 to 50 μg ml^-1^) derivatized in the same way as the samples. The amount of oxidized form of glutathione in the sample was calculated based on the calibration curve prepared for the GSSG standard (Sigma-Aldrich, USA), which was reduced to GSH by added DTT. Eight replicates were used per group.

### ﻿Pigment analysis and measurement of chlorophyll *a* integrity

Photosynthetic pigments were extracted from ca 20 mg of lyophilized lichen samples following the method of Barnes et al. (1992). The samples were washed in CaCO_3_-saturated acetone to remove chlorophyll-degrading substances and those interfering with chlorophyll (six 1-min rinses with 2 ml of medium with vortexing). Pigments were then extracted twice in 3 ml of DMSO with 2.5 mg/ml polyvinylpolypyrrolidone (PVPP) at 65°C for 1 h, followed by centrifugation (10 min at 10,000 × *g*). Absorbance was measured at 665.1, 649.1, 480, 435, and 415 nm, and pigment concentrations were calculated using Wellburn’s formulas (Wellburn 1994). The pheophytinisation quotient related to chlorophyll degradation was calculated from the absorbance ratio at 435 and 415 nm (Garty 2001). All steps were done in semi-dark conditions to prevent chlorophyll degradation. Eight replicates were used per group.

### ﻿Photosynthetic efficiency

Lichen thalli were stored at -30°C directly after the X-ray experiment and kept frozen until fluorescence measurements. The respective portions of lichen were unwrapped directly before the measurements, placed on Petri dishes with a paper layer, and thawed at room temperature (2–3 min). Then, they were thoroughly sprayed with tap water and incubated in darkness for 5 min; transferred to other Petri dishes without the paper layer, and further incubated until reaching a total incubation time of 15 min, before being used for fluorescence imaging measurements. The lichen samples were then kept hydrated by placing them on wet filter paper to prevent water loss for the next 24 h in dim, scattered light. Fluorescence measurements were performed after 210 min and 24 h from lichen watering. Before each measurement, samples were dark-adapted for 10 min; image and setting optimization was performed before darkening.

Chlorophyll fluorescence imaging was performed using a pulse-modulated Open Fluor-Cam FC 800-O/1010 fluorimeter and analyzed using FluorCam7 software (PSI, Drasov, Czech Republic). The modified ‘QuenchingAct2’ protocol was used according to (Osyczka and Myśliwa-Kurdziel 2023). The saturating pulse intensity was 3700 µmol photons m^−2^ s^−1^. The intensity of actinic white light was 900 µmol photons m^−2^ s^−1^ for *C.aculeata* and 1730 µmol photons m^−2^ s^−1^ for *D.muscorum*. These values were previously found to be saturating for each respective species. The following parameters were selected for analysis: the maximum PSII quantum yield (QY_max), an effective PSII quantum yield (QY), and a non-photochemical quenching (NPQ), each measured after 4.2 min of respective actinic light illumination. All measurements were performed with the shutter set to “1” and sensitivity adjusted to 20–40%. Six to eleven replicates were used at each measurement time.

### ﻿Statistical analysis

Two-way analyses of variance (two-way ANOVA; p < 0.05) were performed to assess the effect of lichen species and the experimental group on lichen physiological and biochemical parameters. The significance of differences between particular experimental groups was then verified with Tukey’s HSD post-hoc tests (p < 0.05). The same analyses were run to test the effect of the experimental group and time from hydration on the parameters related to photosynthetic efficiency. Student’s t-tests (p < 0.05) were performed to test the significance of differences in concentrations of secondary metabolites in *D.muscorum*, and cysteine concentrations in *C.aculeata*, between experimental and control groups. Before conducting these analyses, the normality of group distributions was assessed using the Kolmogorov-Smirnov test. The Levene and Brown-Forsythe tests were employed to confirm the homogeneity of variances. When required, a Box-Cox transformation was applied. Statistical analyses were performed using STATISTICA 13 (TIBCO Software Inc., Palo Alto, CA, USA).

## ﻿Results

### ﻿Morphological and anatomical traits

**Figure 2.**
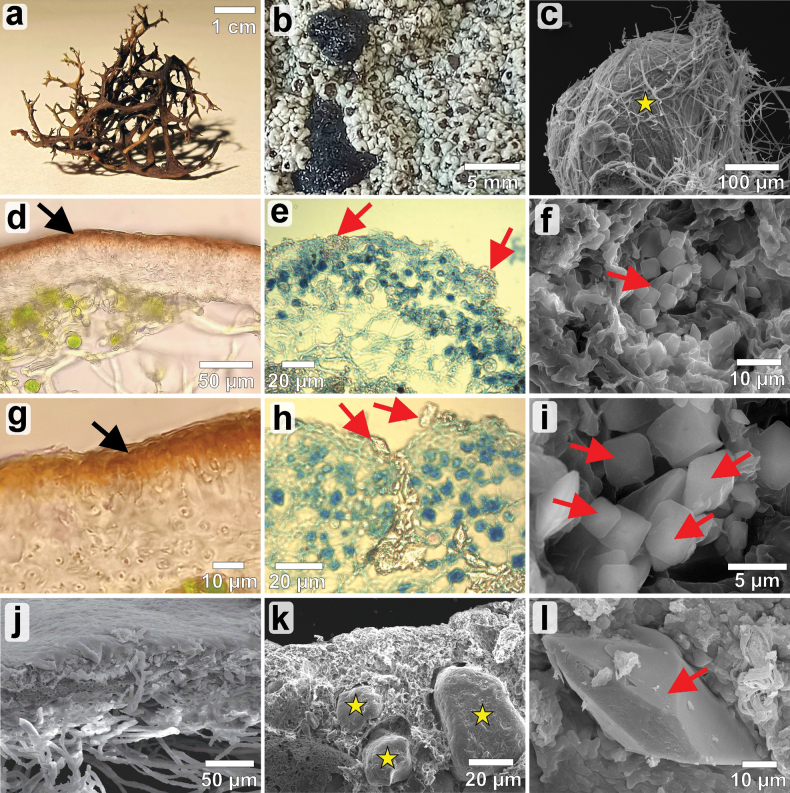
Morphological and anatomical characteristics of *Cetrariaaculeata* (**a, d, g, j**) and *Diploschistesmuscorum* (**b, c, e, f, h, i, k, l**): **a, b** lichen thallus; **d, e, g, h** thallus cross sections in light microscope; **j, k** thallus cross-section in SEM; **c** grain of quartz sand surrounded by fungal hyphae; **f, i, l** calcium oxalate crystals on thallus surface. Black arrows indicate melanin pigments, red arrows indicate calcium oxalate crystals and yellow asterisks indicate grains of quartz sand trapped inside the thallus.

Cross sections of *C.aculeata* thalli showed a cortex layer with an accumulation of melanin pigments in the upper part (Fig. 2d). Part of the cortical layer underneath contained conglutinated fungal cells in a gelatinous matrix (Fig. 2g, j). The algal layer was distributed more or less regularly below the cortex, but the areas without algal cells were also present. The medulla layer was lax and included sparse fungal hyphae located below the algal layer. The surface of the thallus was smooth without distinct ultrasculpture (Fig. 2j).

The cortex layer of *D.muscorum* was not well distinguished but contained an epinecral layer. The algal layer was of varying thickness and the medulla was lax (Fig. 2e, h). Numerous grains of quartz sand were trapped inside the thallus and surrounded by fungal hyphae (Fig. 2c, k; Suppl. material 1: fig. S2). We recorded the presence of numerous calcium oxalate crystals on the thallus surface (Fig. 2e, f, i, l; Suppl. material 1: fig. S2) and inside the thallus (Fig. 2h).

### ﻿Vitality assay and oxidative stress

**Figure 3.**
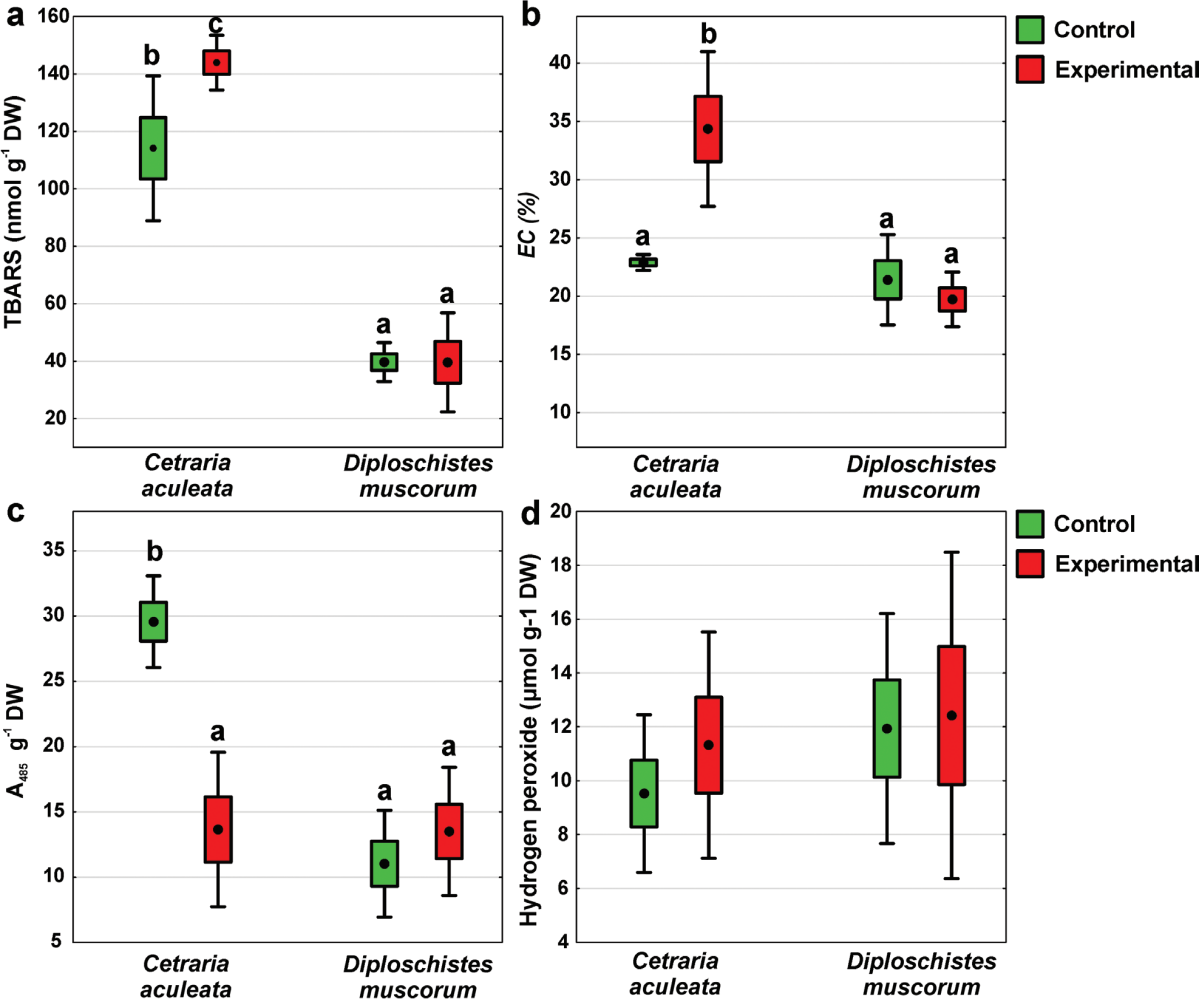
Physiological parameters of *C.aculeata* and *D.muscorum* samples representing control and experimental groups (dot = mean, box = SE, whisker = 95% confidence interval; n = 8): **a** membrane lipid peroxidation (TBARS = Thiobarbituric Acid Reactive Substances), **b** cell membrane integrity (EC = relative electrical conductivity), **c** dehydrogenase activity (A_485_ = absorbance of formazan at 485 nm) and **d** hydrogen peroxide level. The different lowercase letters relate to significant interaction effect and indicate significant differences (p < 0.05). For details see Suppl. material 1: table S1.

Regarding TBARS concentrations, the highest values were observed in *C.aculeata* in the experimental group, which differ significantly from the remaining groups (Fig. 3a; Suppl. material 1: table S1). Contrarily, the lowest TBARS concentrations were found in *D.muscorum* both in the control and experimental group and they did not differ significantly from each other (Fig. 3a). Similar results were observed for cell membrane integrity (Fig. 3b; Suppl. material 1: table S2). Significantly, the highest EC values were recorded in *C.aculeata* in the experimental group while the remaining groups did not differ significantly from each other (Fig. 3b). The highest dehydrogenase activity was observed in *C.aculeata* control group, which differed significantly from the other groups (Fig. 3c; Suppl. material 1: table S1). In contrast, no significant differences were observed among the remaining groups. Regarding hydrogen peroxide level no significant differences were found (Fig. 3d; Suppl. material 1: table S1).

### ﻿Antioxidant activity

**Figure 4.**
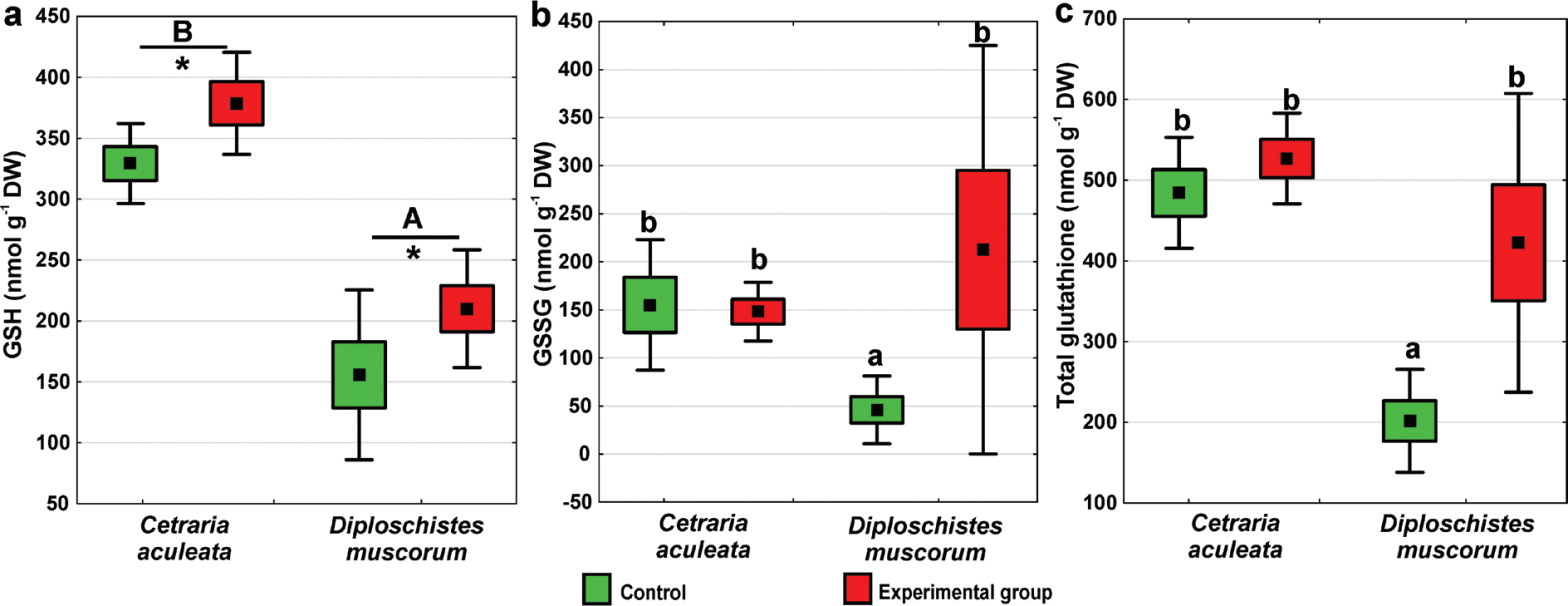
Glutathione concentrations in *C.aculeata* and *D.muscorum* samples representing control and experimental groups (square = mean, box = SE, whisker = 95% confidence interval, for *C.aculeata* n = 8, for *D.muscorum* n = 6): **a** GSH concentration, **b** GSSG concentration, and **c** total glutathione concentration. The different letters above the bars indicate significant differences (p < 0.05). Lowercase letters indicate significant interaction, and capital letters indicate the significant effect of lichen species. The asterisk indicates a significant effect of the experimental group. For details see Suppl. material 1: table S1.

Regarding GSH concentration, both lichen species and the experimental group were found to be significant factors (Suppl. material 1: table S1). *Cetrariaaculeata* had significantly higher GSH concentrations than *D.muscorum*, and in both species, higher concentrations were found in the experimental group compared to the control (Fig. 4a). As regards GSSG, the lowest concentration was observed in the *D.muscorum* control group, while remaining groups did not differ significantly from each other (Fig. 4b; Suppl. material 1: table S1). The same trend was observed for total glutathione level (Fig. 4c; Suppl. material 1: table S1). The ratio of GSH/GSSG in experimental samples of *D.muscorum* was on average more than 3 times lower (2.4) than in control samples (7.3), which corresponds to the increased percentage of GSSG to the total glutathione (42.5±24.5) in experimental samples compared to the control ones (23.0±15.6). Regarding *C.aculeata*, the values of the GSH/GSSG ratio were similar in the experimental (2.8) and control group (3.1). Cysteine concentration in *C.aculeata* thalli was significantly higher in the experimental group compared to the control (Suppl. material 1: fig. S3). The level of cysteine in *D.muscorum* thalli was too low to be determined.

### ﻿Sugar alcohols

**Figure 5.**
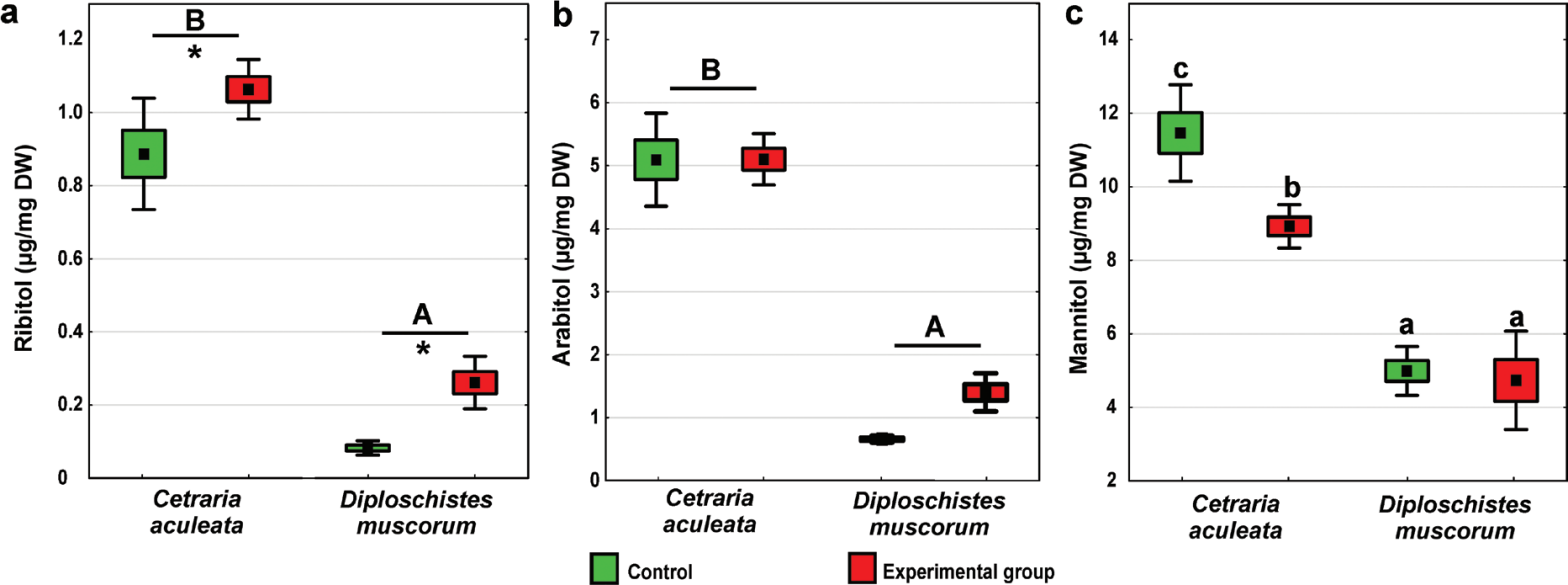
Sugar alcohol concentrations in *C.aculeata* and *D.muscorum* samples representing experimental and control groups (square = mean, box = SE, whisker = 95% confidence interval, n = 8): **a** ribitol concentration, **b** arabitol concentration, and **c** mannitol concentration. The different letters above the bars indicate significant differences (p < 0.05). Lowercase letters indicate a significant interaction, capital letters indicate the significant effect of lichen species. The asterisk indicates a significant effect of the experimental group. For details see Suppl. material 1: table S1.

Both lichen species and the experimental group significantly affected ribitol concentrations (Suppl. material 1: table S1). *Cetrariaaculeata* had significantly higher concentrations than *D.muscorum*, and in both species higher concentrations were found in experimental groups compared to control groups (Fig. 5a). As regards arabitol, only the effect of lichen species was significant (Suppl. material 1: table S1), and *C.aculeata* had significantly higher values compared to *D.muscorum* (Fig. 5b). The highest mannitol concentrations were recorded in *C.aculeata* control group, differing significantly from the other groups. The lowest values were observed in both *D.muscorum* groups, which did not differ significantly from each other (Suppl. material 1: table S1; Fig. 5c).

### ﻿Secondary metabolites

**Figure 6.**
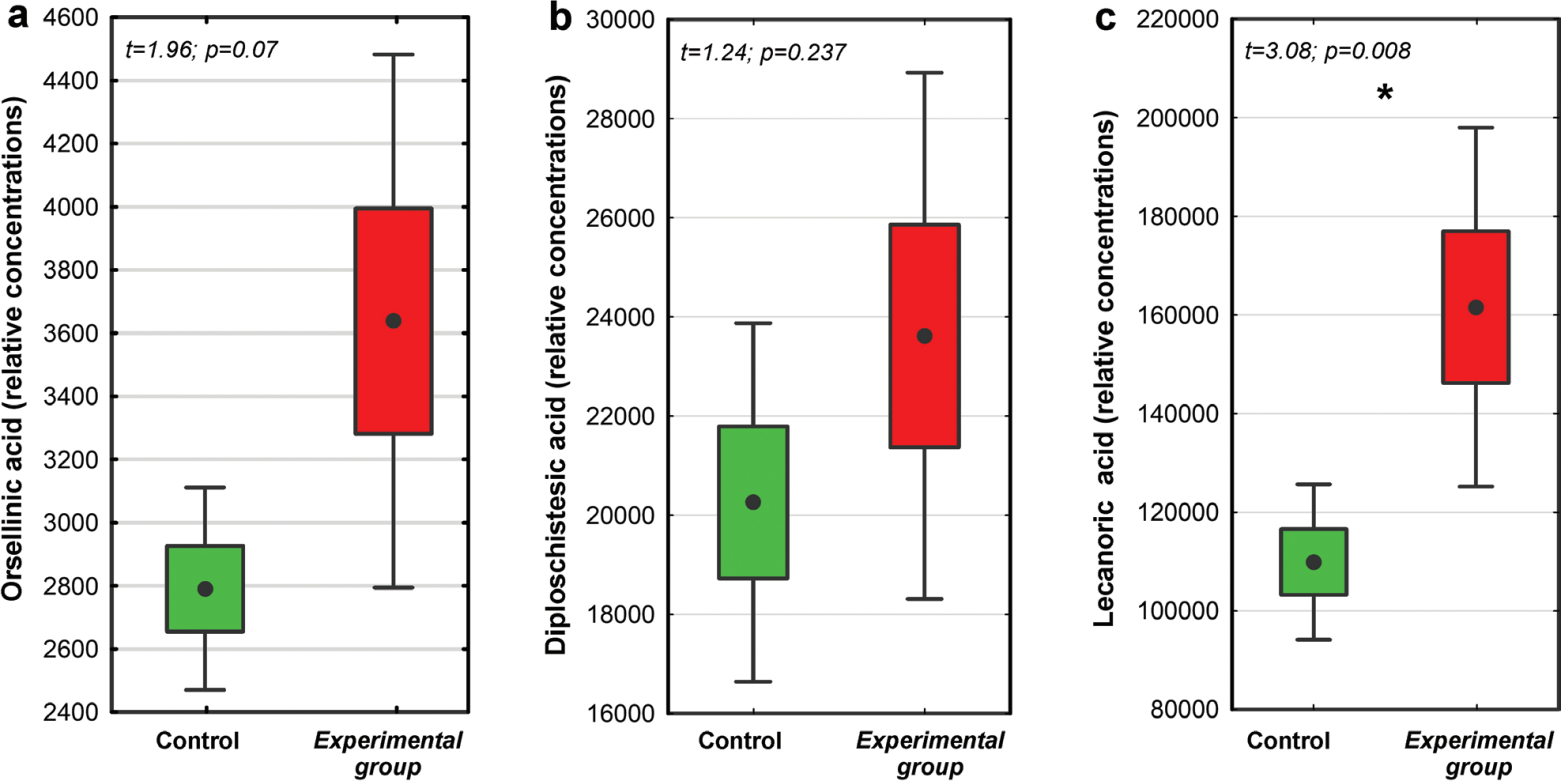
The relative concentrations of secondary metabolites **a** orsellinic acid **b** diploschistesic acid and **c** lecanoric acid in *D.muscorum* samples representing control and experimental groups (dot = mean, box = SE, whisker = 95% confidence interval, n = 8). Student’s t-test results are provided above the graphs (p < 0.05).

Regarding secondary metabolites in *D.muscorum* higher concentrations were recorded in the experimental group; however, only lecanoric acid concentrations were significantly higher in the experimental group compared to the control (Fig. 6c).

### ﻿Photosynthetic pigments

**Figure 7.**
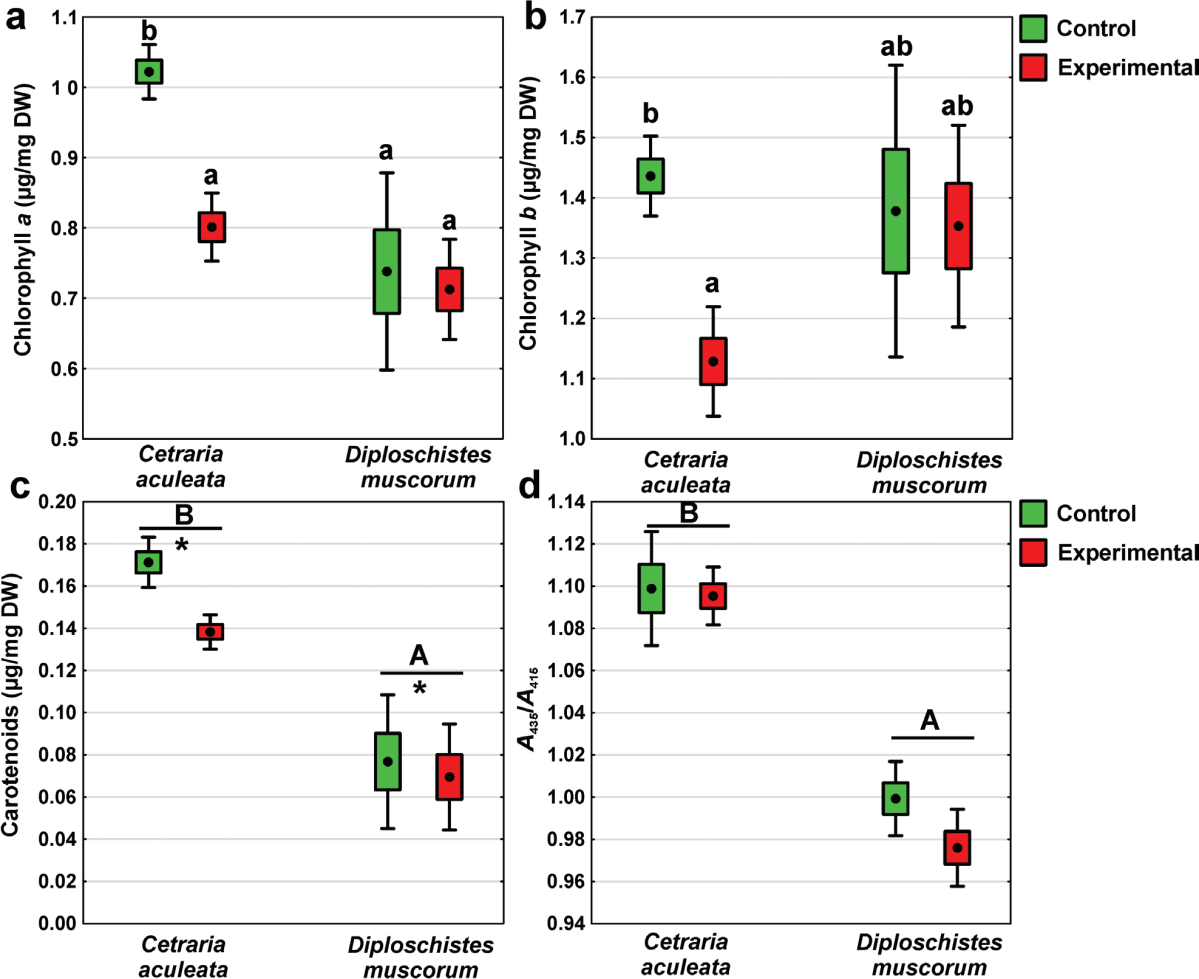
Parameters related to photosynthetic pigments of *C.aculeata* and *D.muscorum* samples representing control and experimental groups (dot = mean, box = SE, whisker = 95% confidence interval; n = 8): **a** chlorophyll *a* concentration, **b** chlorophyll *b* concentration, **c** carotenoids concentration and **d** A_435_/A_415_ ratio. The different letters above the bars indicate significant differences (p < 0.05). Lowercase letters indicate a significant interaction, capital letters indicate the significant effect of lichen species. The asterisk indicates a significant effect of the experimental group. For details see Suppl. material 1: table S1.

The highest chlorophyll *a* concentrations were observed in *C.aculeata* control group, which differed significantly from the remaining groups (Fig. 7a; Suppl. material 1: table S1). In contrast, no significant differences were observed among the remaining groups. Concerning chlorophyll *b* concentration, a significant decrease in the experimental group compared to the control was observed in *C.aculeata* (Fig. 7b; Suppl. material 1: table S1). Significantly higher carotenoid concentrations were observed in *C.aculeata*, as well as in the control groups compared to experimental groups in both species (Fig. 7c; Suppl. material 1: table S1). Regarding A_435_/A_415_ only the effect of lichen species was significant (Suppl. material 1: table S1), with *C.aculeata* reaching higher values (Fig. 7d).

### ﻿Photosynthetic efficiency

**Figure 8.**
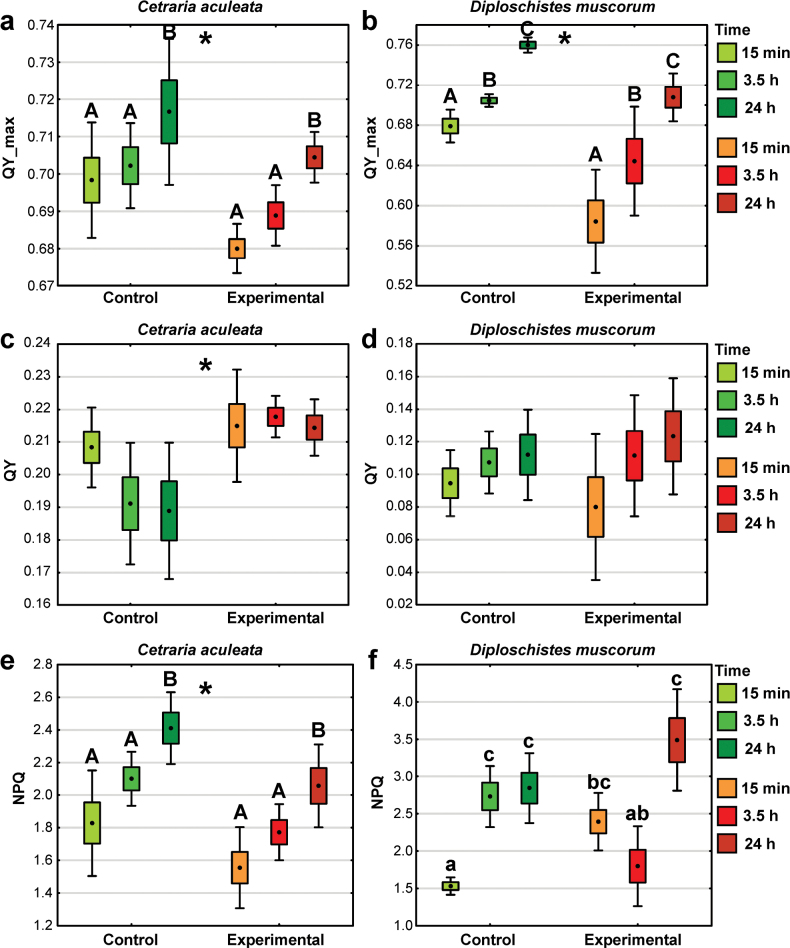
Chl *a* fluorescence parameters of *C.aculeata* and *D.muscorum* samples representing control and experimental groups in relation to time after hydration (dot = mean, box = SE, whisker = 95% confidence interval; n = 6–11): **a, b** QY_max – maximum PSII quantum yield in a dark-adapted sample; **c, d**QY – the effective PSII quantum measured in light; **e, f**NPQ – the non-photochemical fluorescence quenching in light state. The different letters above the bars indicate significant differences (p < 0.05). Lowercase letters indicate a significant interaction, capital letters indicate the significant effect of time. The asterisks indicate a significant effect of the experimental group. For details see Suppl. material 1: table S2.

Regarding QY_max (maximum PSII quantum yield), both the experimental group and the time after hydration following the experiment were found to be significant factors for both lichen species (Suppl. material 1: table S2). It increased with time being the highest after 24 h from thallus hydration and experimental samples had significantly lower values of this parameter than the control samples (Fig. 8a, b; Suppl. material 1: figs S4, S5). For both control and experimental samples of *C.aculeata*, just 15 min of hydration resulted in the QY_max of about 95% of the QY_max reached at 24 h post-hydration. QY_max recovery was relatively slow in *D.muscorum*, especially in experimental samples. The time after hydration did not affect significantly QY; however, it was significantly higher in experimental samples of *C.aculeata* compared to the control. As regards *D.muscorum*, neither time nor experimental group was significant, and QY remained low in all cases (Fig. 8d). NPQ in *C.aculeata* increased significantly with time and experimental samples had significantly lower values than the control (Fig. 8e). Regarding *D.muscorum*, the highest values ​​were recorded in the control group after 3.5 h and 24 h and in the experimental group after 24 h from thallus hydration (Fig. 8f; Suppl. material 1: table S2).

## ﻿Discussion

Ionizing radiation on Mars’s surface is one of the limiting factors for life survival and habitability (Horneck et al. 2010). So far, many extremophiles have shown resistance to ionizing radiation in dehydrated, anhydrobiotic state (see Dadachova and Casadevall 2008). Since ionizing radiation and water-rich environments are highly detrimental, it is crucial to assess whether metabolically active organisms can function under Mars-like conditions. Previous studies showed that free-living extremophilic fungi in a metabolically active state are much more susceptible to ionizing radiation than in a dehydrated state (Pacelli et al. 2017, 2018; Cassaro et al. 2025). However, these experiments were conducted in terrestrial conditions, where the only stress factor was ionizing radiation. Overall, our study demonstrated that metabolically active *D.muscorum* exhibited significantly greater resistance to simulated Mars-like conditions compared to *C.aculeata*. This proves that the response strongly depends on the species. However, interpretation is challenging because metabolically active organisms are potentially more vulnerable to radiation but can also activate repair mechanisms to mitigate damage. Fungal and algal cells have evolved various adaptations including protective enzymes, antioxidants, and polyols, which mitigate the harmful effects of increased ROS (Kranner et al. 2005, 2008). Secondly, the studied species differ in morphological/anatomical and biochemical traits that may provide them protection against the negative effects of ionizing radiation.

### ﻿Oxidative stress and lichen responses

The surface of Mars is constantly exposed to high levels of both UV and ionizing radiation because this planet has thin atmosphere and does not have a global magnetic field (Acuña et al. 1998). Ionizing radiation can lead to both direct and indirect harm to biological molecules (Nelson 2003). Direct action occurs through ionization and excitation of electrons in atoms. Equally dangerous are indirect radiation damages caused by ROS that are produced by the interaction of radiation with cellular water in a process called water radiolysis (Reisz et al. 2014). The desiccated state of lichens, with water content below 5% without cytoplasmic bulk water (Kranner et al. 2008), significantly minimizes the damage caused by ionizing radiation (Cassaro et al. 2025). Our study focused on hydrated and metabolically active lichens and showed how they could potentially cope with functioning on Mars, rather than just surviving in a desiccated state. This is crucial since water radiolysis is a major source of free radicals causing cellular oxidative damage (Sharma et al. 2012). Our results showed different responses of both studied species. Regarding *C.aculeata*, we observed a high level of oxidative stress, as reflected by a high level of membrane lipid peroxidation and damage, decreased dehydrogenase activity, and slightly elevated level of H_2_O_2_ accumulated in cells (Fig. 3). This suggests that in this species the intracellular ROS concentration increased above the level that could be handled by antioxidant defense systems. The severe impact on the mitochondrial respiratory chain in *C.aculeata* may stem from radiation-induced damage to both hydrophobic and hydrophilic parts of membrane lipids as was shown in a model membrane (Shadyro et al. 2002). The decrease of dehydrogenase activity by up to half after the experiment confirms the reduced viability of the mycobiont and suggests that the mitochondria membranes may be the main targets for X-ray-induced ROS. On the other hand, we did not observe clear symptoms of oxidative stress in *D.muscorum*, which in turn indicates its high resistance and/or effective defense against oxidative stress. We recognize, however, that this effect may not be solely attributable to X-rays but could have resulted from the combined influence of all the factors introduced to simulate the Mars atmosphere. In active lichens, defense mechanisms and increased antioxidants may reduce oxidative stress, similar to plants exposed to ionizing radiation (see Gudkov et al. 2019). Thanks to this, repair mechanisms can effectively limit the negative effects. The only study to date on metabolically active lichens exposed to X-rays (up to 100 Gy) found that photobiont photosynthetic efficiency was not affected (Brandt et al. 2017). Nevertheless, nothing was known about the remaining physiological/biochemical parameters of both symbiotic partners. The high resistance of *D.muscorum* could be attributed to the production of secondary metabolites, i.e., lecanoric and orsellinic acids, known for their strong antioxidant activity (Lopes et al. 2008). After the experiment, their concentrations were increased, which could indicate their role in protection against oxidative stress. A similar effect was observed in vascular plants (Taheri et al. 2014), even during a few minutes of exposure to ionizing radiation (Dixit et al. 2010).

Lichens are known to have powerful detoxification systems including glutathione – an essential non-enzymatic antioxidant (Kranner and Lutzoni 1999). It occurs in cells primarily in reduced form (GSH) and in lower amounts in an oxidized form (GSSG) (Wonisch and Schaur 2001). Experimental samples of *D.muscorum* had significantly increased levels of total glutathione. Similarly, plants showed increased glutathione levels after ionizing radiation exposure (e.g., Aly and El-Beltagi 2010; van de Walle et al. 2016). In lichens, under normal physiological conditions, GSSG makes up approximately 10–23% of the total glutathione in hydrated thallus (Kranner 2002), as reflected in our results from the control group of *D.muscorum* (Fig. 4b). However, in experimental samples, an increased amount of GSSG in relation to GSH was pronounced (GSSG comprised up to 42.5% of total glutathione). Since under stress conditions, ROS can oxidize GSH to GSSG (Zechmann et al. 2008; Noctor et al. 2012), it seems that a large part of GSH scavenged ROS, being oxidized to GSSG, thereby protecting membrane lipids and enzyme proteins from peroxidation. Consequently, no significant changes in TBARS levels, cell membrane integrity, or dehydrogenase activity were observed in *D.muscorum* (Fig. 3). This can be considered as an adaptive response, with glutathione playing a crucial role in reducing oxidative stress, thereby enhancing *D.muscorum* stress tolerance. Similarly, research on the effect of low doses of gamma radiation on *Lemnaminor* indicated non-enzymatic antioxidants as key to protecting cells from ROS damage (Xie et al. 2019). Importantly, our results proved that during the experiment in simulated Mars-like conditions, *D.muscorum* was capable of performing metabolic processes at least for part of the experiment and effectively activate defense mechanisms. This indicates its ability to maintain a balance between the triggering of the damage response and the degree of this damage. In *C.aculeata*, however, elevated oxidative stress and associated damage were not effectively balanced despite increased glutathione and cysteine biosynthesis (the metabolic precursor of glutathione). This demonstrates a considerable imbalance between ROS production and the quenching effect of glutathione.

It is important to highlight that our study involved radiation exposure expected on the Mars surface over one year of strong solar activity, whereas in an actual Mars surface environment, life forms would experience prolonged, chronic radiation exposure. Simulating long-term space radiation exposure by delivering an equivalent dose over a short period is a well-established method in astrobiology (e.g., Horneck et al. 2010; Brandt et al. 2017; Cassaro et al. 2025). Although acute and chronic radiation exposure may lead to different biological effects, this approach remains one of the most viable ways to investigate the impact of radiation on lichens. In this context, our findings lay the foundation for future studies, including long-term exposure experiments on the Mars surface.

### ﻿Effect of low-pressure Mars-like CO_2_ atmosphere

Apart from X-ray radiation, the exposure to a low-pressure Mars-like CO_2_ atmosphere could have affected symbiont metabolism. The fungal component primarily relies on aerobic respiration, which requires oxygen to break down carbohydrates and produce energy, thus efficiently metabolizing. Previous studies showed that aerobic bacteria and fungi can survive and metabolize in a CO_2_-dominant atmosphere, but their activity and growth were rather limited (e.g. Schuerger and Nicholson 2006). Although we cannot estimate the long-term effects of the fungal partner being kept under such conditions due to our study design, the increased glutathione production and increased GSSG/GSH ratio in the experimental samples indicate active metabolic processes during a certain part of the 5-hour experiment. Importantly, de Vera et al. (2014) revealed that lichen photosynthetic partners can adapt physiologically to live on Mars based on photosynthetic activity assessment during a 34-day exposure to Mars-like conditions. Therefore, it can be assumed that lichen algae may still produce oxygen, which the fungal partner could potentially utilize. The question is whether the produced oxygen could fully support the fungal partner’s respiration needs, and this aspect requires further research to determine whether the entire symbiosis is able to sustain long-term metabolism and energy balance.

### ﻿Sugar alcohols

Sugar alcohols support metabolism and enhance stress tolerance in lichens (Pichler et al. 2023) by providing energy for growth, facilitating the solubilization of enzymes during freezing, and stabilizing cellular structures such as membranes and proteins during dehydration (Palmqvist 2000; Kranner et al. 2022). Ribitol is produced by the photobiont and exported to the fungus, where it is converted into arabitol and mannitol (Eisenreich et al. 2011). Interestingly, we found an increased amount of ribitol in both species after exposure to experimental conditions. This may be related to a change in the rate of leakage of ribitol from the alga to the fungus or/and disruption of metabolic processes of the fungus, which converts ribitol to arabitol/mannitol. Since in both the experimental and control conditions, there was no access to photosynthetically active radiation, photosynthesis should not occur, thus we initially suppose a reverse trend or at least an unchanged polyol level. In control conditions, ribitol was probably normally transported to the fungus and converted to arabitol and mannitol (Honegger 1991), which led to a lower ribitol concentration; whereas during exposure to experimental conditions, these processes could have been disturbed. This explanation is supported by the fact that under normal conditions the ribitol is rapidly transferred to the mycobiont and irreversibly metabolized into different forms (Spribille et al. 2022).

### ﻿Photobiont responses

Photosynthetic pigments are sensitive to ionizing radiation in plants (Gudkov et al. 2019). After low-dose exposure, chlorophyll levels either remained unchanged or increased, likely due to enhanced chlorophyll biosynthesis through enzyme activation, a phenomenon known as radiation hormesis (Jan et al. 2011; Volkova et al. 2022). On the other hand, exposure to high doses of gamma radiation caused a considerable decrease in both chlorophyll and carotenoids (e.g., Goh et al. 2014; Hong et al. 2014). Linczerski et al. (2023) observed a decrease in carotenoid content in plant *Astrophytum* spp. after exposure to an X-ray dose of 50 Gy. We also observed a significant decrease in chlorophyll and carotenoids in *C.aculeata* exposed to the same X-ray dose. This effect may result from pigment degradation due to the oxidation by radiation-induced ROS (cf Dartnell et al. 2011). Membrane and protein ROS-induced damage, including the disruption of photosynthetic antenna complexes hosting the majority of chlorophylls and carotenoids, cannot be excluded either. Oxidative stress likely contributed to the decrease in photosynthetic pigments in *C.aculeata*, as we observed increased oxidative stress and cell membrane damage (Fig. 3), potentially causing lipid peroxidation in the chloroplast membrane. On the other hand, the level of the A_435_/A_415_ ratio, an indicator of chlorophyll integrity (Ronen and Galun 1984) remained unchanged in *C.aculeata* (Fig. 7d). This means that the level of phaeophytin, the chlorophyll breakdown product (Tanaka and Ito 2024), did not increase. This observation suggests a complex interaction between chlorophyll degradation and the physiological responses of *C.aculeata* exposed to experimental conditions. While chlorophyll levels decline, the rate of degradation is somehow balanced by the simultaneous degradation of pheophytin, maintaining a constant A_435_/A_415_ ratio. This indicates that pigment degradation is a regulated process, potentially reflecting lichen’s adaptive strategies to mitigate environmental stress. The inactivation of a chlorophyll-synthesizing system by X-rays has been already described in free-living algae (Kohn et al. 1967) and reported in vascular plants (e.g., Saha et al. 2010). On the contrary, *D.muscorum* showed resistance, with chlorophyll and carotenoid levels unchanged under experimental conditions.

Photobiont photosynthesis turned out to be a metabolic process not particularly sensitive to X-ray exposure in the Mars-like atmosphere under dark conditions. Immediately, after thawing and rehydration, both *C.aculeata* and *D.muscorum* were photosynthetically active, with recovery appearing faster in the former species. The samples reached the maximum quantum yield corresponding to physiologically healthy thallus (Jensen and Kricke 2002) already after 15 min for *C.aculeata*. Nevertheless, it is worth noting that the respective values of this parameter were significantly lower in the experimental samples compared to the control. On the other hand, the steady-state PSII quantum yield in light (QY), which refers to the efficiency of using the absorbed energy for PSII photochemistry and electron transport, indicates that the absorbed light energy is effectively converted into chemical energy in the light photosynthetic reactions in experimental samples. The non-photochemical quenching (NPQ) increased after thallus hydration and reached the highest values after 24 h both in control and experimental samples; however, the kinetics of NPQ differ between control and experimental groups in *D.muscorum*. Such results indicate that experimental samples have a similar ability to balance light absorption and utilization for photosynthesis as control samples (Horton and Ruban 1992; Goss and Lepetit 2015). The lack of major photosynthetic disturbances may be due to lichens’ ability to quickly resynthesize chlorophyll after hydration, despite a significant decrease in chlorophyll content, as observed in *C.aculeata*. Such attribute of lichens was also shown by Kranner et al. (2003) who demonstrated that chlorophyll concentrations significantly increase during rehydration within 10 min, regardless of prior desiccation or chlorophyll degradation levels.

### ﻿Potential adaptive traits of lichens

Several adaptive strategies related to anatomy, morphology, and biochemistry have been identified, enhancing lichen resistance to extraterrestrial conditions (Meeßen et al. 2013). Therefore, in the context of further astrobiological research, it seems crucial to search for more such features. In our study, we deliberately selected two lichen species with traits that could potentially be important in the context of resistance to Mars-like conditions. We found that although *C.aculeata* accumulates melanins in the cortex layer of the thallus, they do not provide optimal protection against ionizing radiation in Mars-like conditions as was previously revealed in non-lichenized fungi (Onofri et al. 2008). Melanin pigments are present in fungal species from high-radiation environments on Earth, such as the damaged Chernobyl nuclear reactor (Casadevall et al. 2017). Melanin can absorb a wide spectrum of electromagnetic radiation, including X-rays and gamma rays, giving it a shielding capacity against ionizing radiation that is about half that of lead (Dadachova et al. 2008). Previous studies suggested melanins aid radioprotection by physically shielding and quenching cytotoxic free radicals, as they can scatter or trap photons and electrons (Dadachova et al. 2008; Pacelli et al. 2017, 2018). However, our results do not confirm the antioxidant or shielding function of melanin in this lichenized fungus as we recorded a high level of oxidative stress. This may be related to the very diverse chemical structure of melanin molecules, confirming that the internal chemical composition of melanin may contribute to its radioprotective properties (Dadachova et al. 2008). On the other hand, the high resistance of *D.muscorum* is probably related to several features of its anatomy/morphology. The thallus is strongly attached to the substrate by medullary hyphae, often incorporating small sand particles (Fig. 2c, k). It also has a well-developed pruina on the thallus surface with numerous calcium oxalate crystals (Fig. 2h, f). We suppose that this crystalline deposit may have great protective significance, as it may induce increased cortical absorbance and reflection (Büdel and Scheidegger 1996). While calcium oxalate has a relatively low atomic number, which makes it less effective at absorbing X-rays than heavier elements, the dense crystal deposits on the thallus surface could allow calcium atoms to interact with low-energy X-rays, absorbing part of their energy (see Elbanna et al. 2020). This interaction may help to reduce the amount of radiation penetrating the thallus interior. Moreover, particles of the parent substrate incorporated into the thallus can provide equally effective protection for the fungal hyphae beneath the thallus. The importance of the cortical layer for resistance to extreme factors has been noted in two model lichen species in astrobiology. *Rhizocarpongeographicum* and *Xanthoriaelegans* exposed to outer space with removed cortical layer had significantly greater damage than intact thallus (de la Torre et al. 2010). Finally, extracellular crystalline deposits were observed in *Circinariagyrosa*, suggesting this is a key adaptation of extreme-tolerant lichens to extraterrestrial conditions (Böttger et al. 2014).

## ﻿Conclusions

Our study is the first to demonstrate that the metabolism of the fungal partner in lichen symbiosis was active while being in an environment similar to the surface of Mars in the darkness. We can conclude that *D.muscorum* can withstand in a metabolically active state Mars-like conditions with an X-ray radiation dose of 50 Gy that is expected on the Mars surface over one year of strong solar activity. Consequently, the high doses of X-rays associated with solar flares and SEPs reaching the surface of Mars should not affect the potential habitability of Mars by lichens. We suggest that *D.muscorum* is a promising candidate for further astrobiological research.

## ﻿Additional information

### Adherence to national and international regulations

All the fungal strains used in this study have been legally obtained, respecting the Convention on Biological Diversity (Rio Convention).

### Author ORCIDs

Kaja Skubała https://orcid.org/0000-0003-2241-1081

Karolina Chowaniec https://orcid.org/0000-0001-7532-856X

Mirosław Kowaliński https://orcid.org/0000-0002-3483-5415

Tomasz Mrozek https://orcid.org/0000-0003-4142-366X

Jarosław Bąkała https://orcid.org/0000-0001-5142-9681

Ewa Latkowska https://orcid.org/0000-0001-9220-1210

Beata Myśliwa-Kurdziel https://orcid.org/0000-0002-3361-7934

### Data availability

The data presented in this article were deposited in public repository RODBUK Cracow Open Research Data Repository and are available at the following link: https://doi.org/10.57903/UJ/QQW7OL.

## Supplementary materials

Supplementary material 1 Supplementary figures and tables Data type pdf Explanation note **figure S1.** Diagram showing the construction (**a**) and arrangement (**b**) of the plate on which lichen samples were placed inside the vacuum chamber during the experiment. **figure S2.** The energy-dispersive X-ray spectroscopy (EDX) analysis of grains of quartz sand trapped in the thallus of *Diploschistesmuscorum* (**a**) and calcium oxalates crystals on *D.muscorum* thallus surface (**b**). **figure S3.** The concentration of cysteine in *Cetrariaaculeata* samples representing control and experimental groups (dot = mean, box = SE, whisker = 95% confidence interval, n = 8). The result of Student's t-test is provided above the graph (p < 0.05). **figure S4.** False color fluorescence image (QY_max – maximum PSII quantum yield in a dark-adapted sample) of *Cetrariaaculeata* samples representing control (**a–c**) and experimental (**d–f**) groups in relation to time after hydration: 15 min (**a, d**), 3.5 h (**b, e**) and 24 h (**c, f**). **figure S5.** False color fluorescence image (QY_max – maximum PSII quantum yield in a dark-adapted sample) of *Diploschistesmuscorum* samples representing control (**a–c**) and experimental (**d–f**) groups in relation to time after hydration: 15 min (**a, d**), 3.5 h (**b, e**) and 24 h (**c, f**). **table S1.** The results of two-way analysis of variance (p < 0.05) for the effect of experimental group (control, experimental) and lichen species (*Cetrariaaculeata*, *Diploschistesmuscorum*) on physiological and biochemical parameters of lichen samples. **table S2.** The results of two-way analysis of variance (p < 0.05) for the effect of the experimental group (control, experimental) and time from hydration (15 min, 3.5 h, 24 h) on the parameters related to photosynthetic efficiency in lichen samples. **table S3.** Conditions used in the simulation experiment compared to surface conditions on Mars. https://binary.pensoft.net/file/1295819This dataset is made available under the Open Database License (http://opendatacommons.org/licenses/odbl/1.0/). The Open Database License (ODbL) is a license agreement intended to allow users to freely share, modify, and use this Dataset while maintaining this same freedom for others, provided that the original source and author(s) are credited.Kaja Skubała, Karolina Chowaniec, Mirosław Kowaliński, Tomasz Mrozek, Jarosław Bąkała, Ewa Latkowska, Beata Myśliwa-Kurdziel
