# Supplementary material for: ﻿Ionizing radiation resilience: how metabolically active lichens endure exposure to the simulated Mars atmosphere
Source: IMA Fungus. 2025 Mar 31;16:e145477. doi: 10.3897/imafungus.16.145477 (PMC11976309; doi:10.3897/imafungus.16.145477)
Supplement: Supplementary material 1 — Supplementary figures and tables [file imafungus-16-e145477-s001.pdf]

SUPPLEMENTARY MATERIALS TO THE ARTICLE

Ionizing radiation resilience: how metabolically active lichens endure exposure to the simulated Mars atmosphere

Kaja Skubała[1*], Karolina Chowaniec[1,2], Mirosław Kowaliński[3], Tomasz Mrozek[3], Jarosław Bąkała[3], Ewa Latkowska[4], Beata Myśliwa-Kurdziel[5]

[1] Institute of Botany, Faculty of Biology, Jagiellonian University, Kraków, Poland
[2] Doctoral School of Exact and Natural Sciences, Jagiellonian University in Kraków, Prof. S. Łojasiewicza 11, 30-348, Kraków, Poland
[3] Space Research Centre, Polish Academy of Sciences, Warsaw, Poland
[4] Laboratory of Metabolomics, Faculty of Biochemistry, Biophysics and Biotechnology, Jagiellonian University, Kraków, Poland
[5] Department of Plant Physiology and Biochemistry, Faculty of Biochemistry, Biophysics and Biotechnology, Jagiellonian University, Gronostajowa 7, 30-387 Kraków, Poland
*Correspondence: kaja.skubala@uj.edu.pl

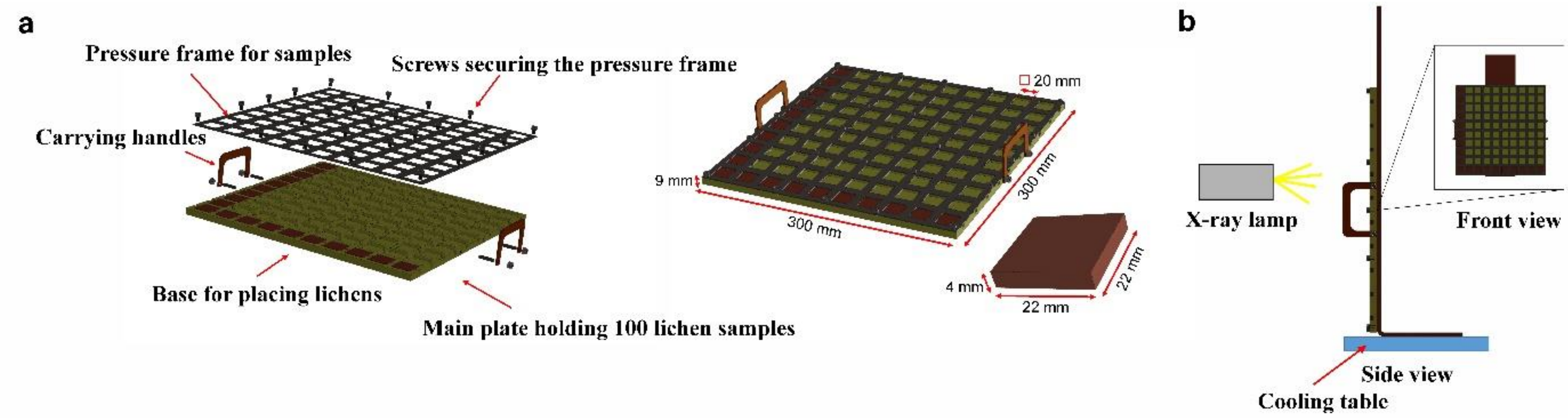

Figure S1. Diagram showing the construction (**a**) and arrangement (**b**) of the plate on which lichen samples were placed inside the vacuum chamber during the experiment.

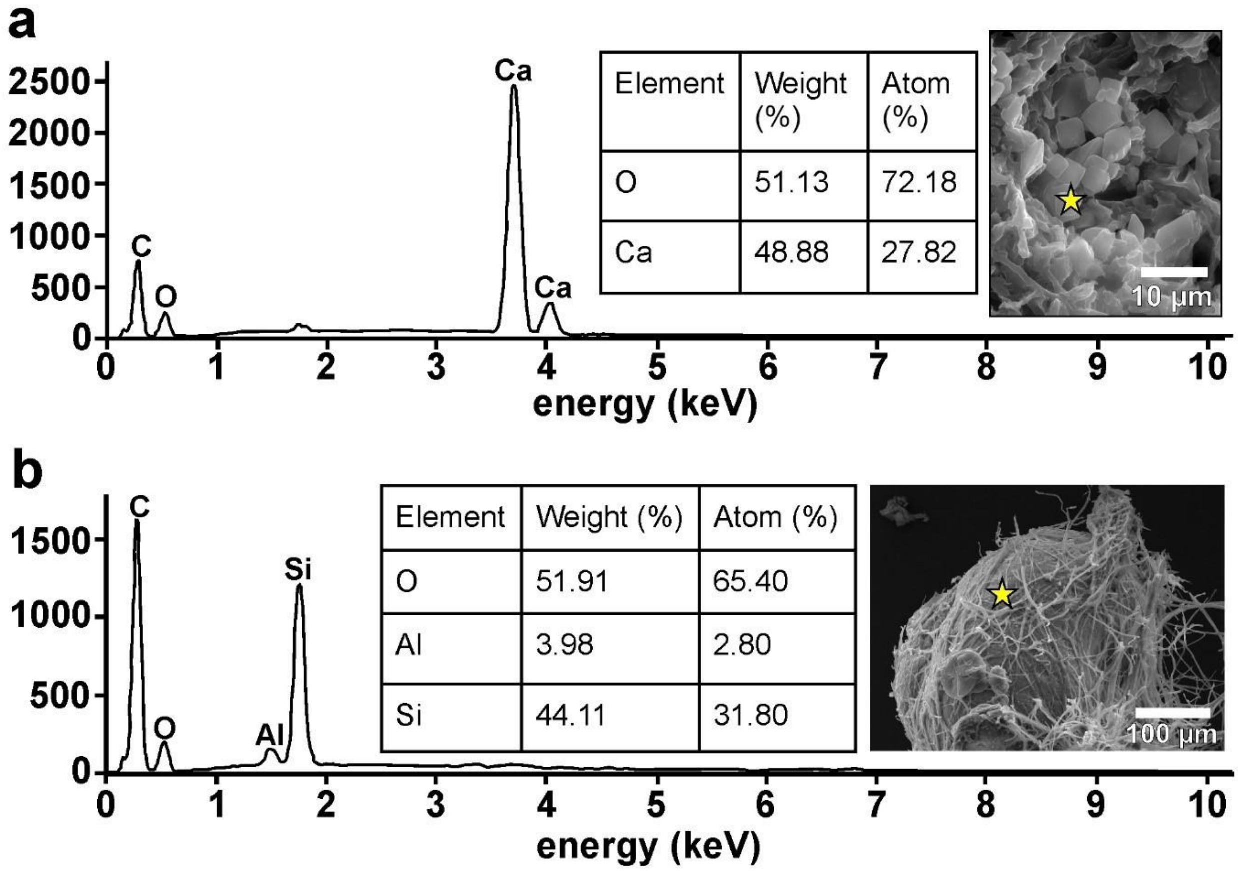

Figure S2. The energy-dispersive X-ray spectroscopy (EDX) analysis of grains of quartz sand trapped in the thallus of *Diploschistes muscorum* (**a**) and calcium oxalates crystals on *D. muscorum* thallus surface (**b**).

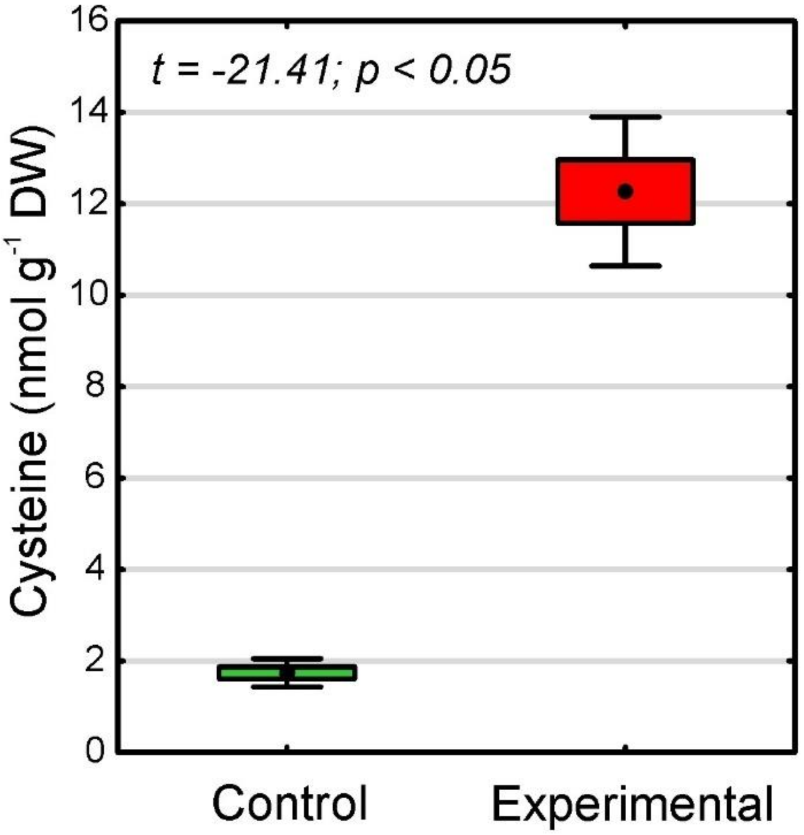

Figure S3. The concentration of cysteine in *Cetraria aculeata* samples representing control and experimental groups (dot = mean, box = SE, whisker = 95% confidence interval, n = 8). The result of Student's t-test is provided above the graph ($p < 0.05$).

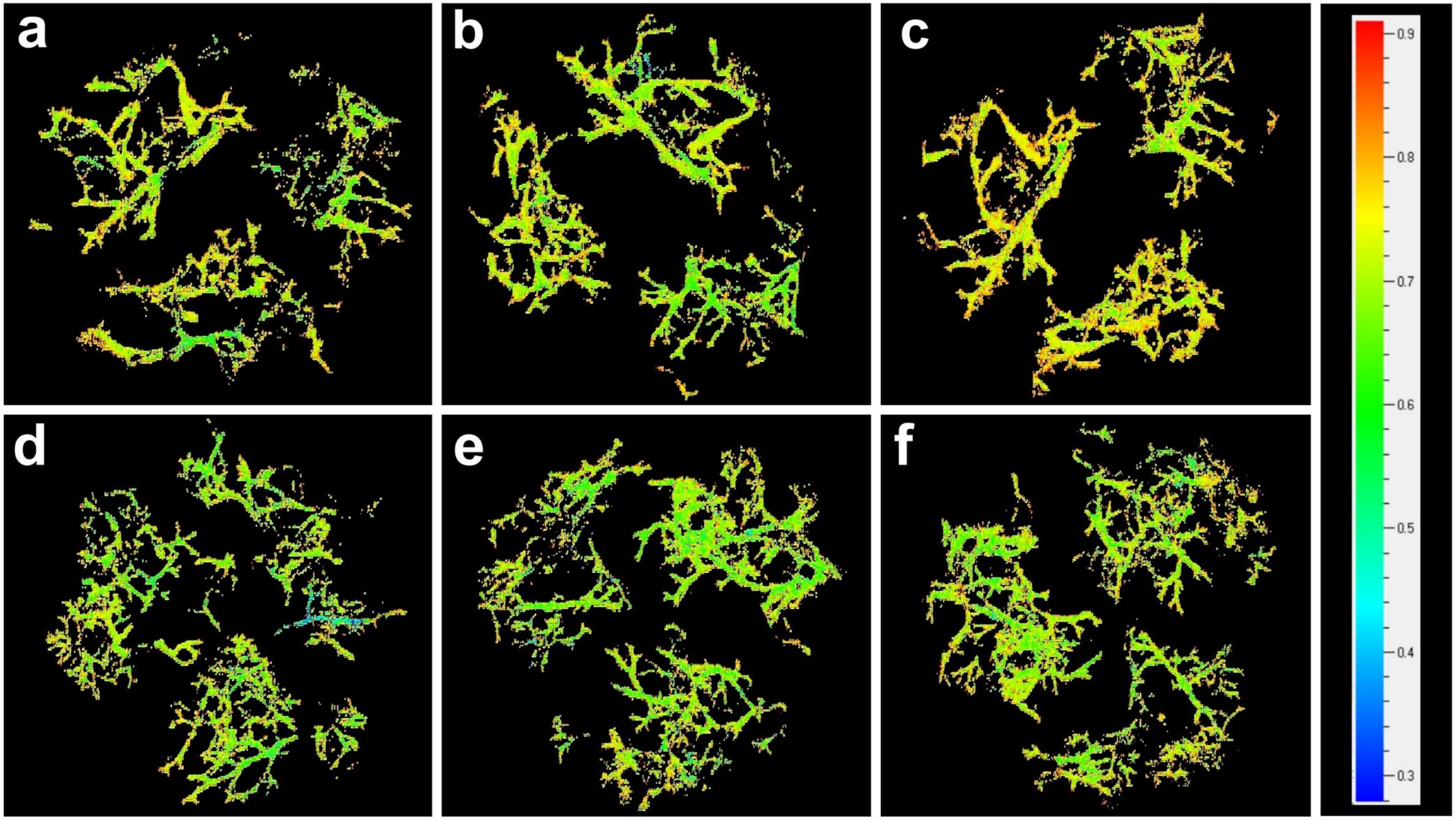

Figure S4. False color fluorescence image (QY_max – maximum PSII quantum yield in a dark-adapted sample) of *Cetraria aculeata* samples representing control (**a-c**) and experimental (**d-f**) groups in relation to time after hydration: 15 min (**a, d**), 3.5 h (**b, e**) and 24 h (**c, f**).

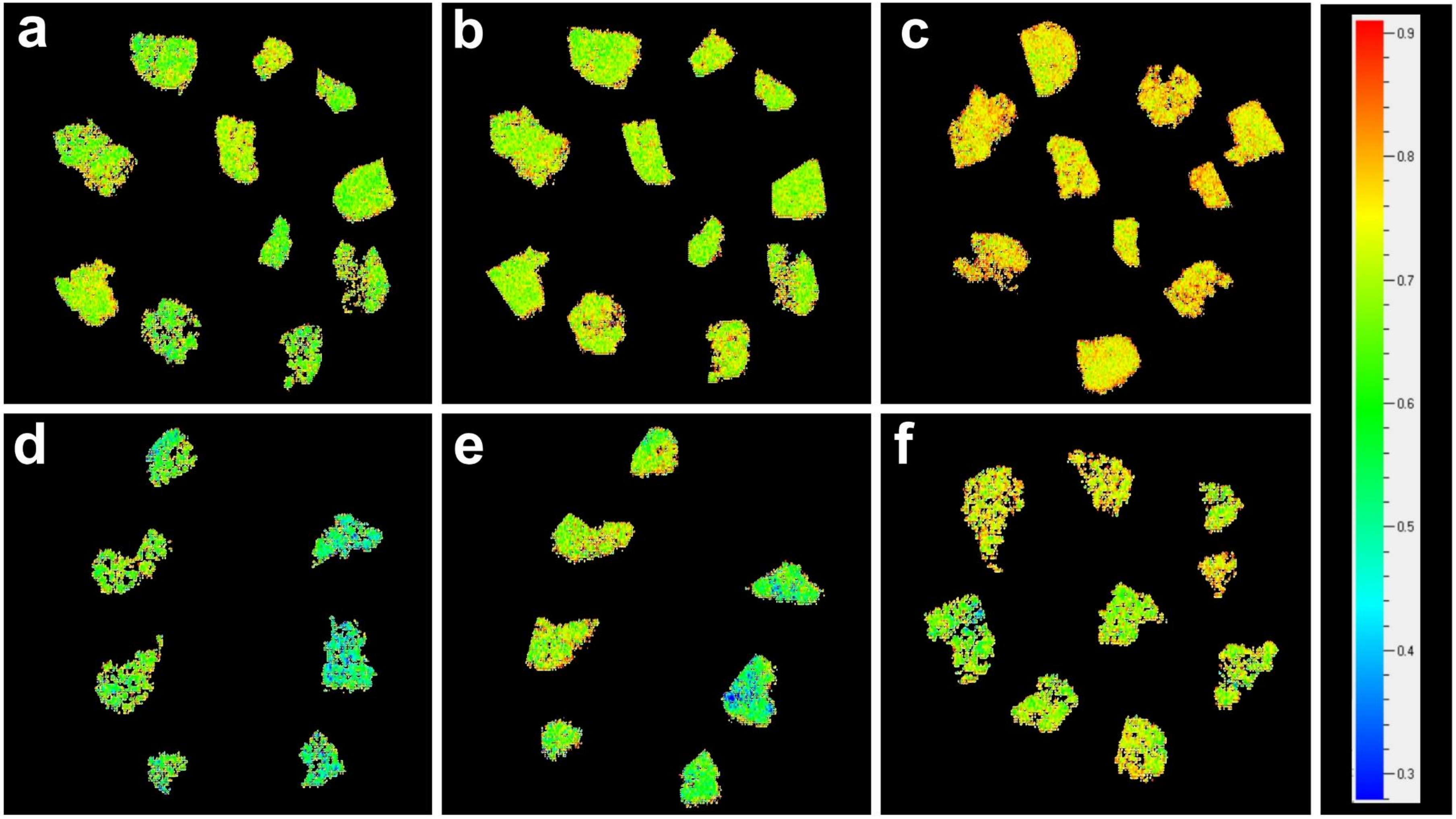

Figure S5. False color fluorescence image (QY_max – maximum PSII quantum yield in a dark-adapted sample) of *Diploschistes muscorum* samples representing control (**a-c**) and experimental (**d-f**) groups in relation to time after hydration: 15 min (**a, d**), 3.5 h (**b, e**) and 24 h (**c, f**).

Table S1. The results of two-way analysis of variance ($p < 0.05$) for the effect of experimental group (control, experimental) and lichen species (*Cetraria aculeata*, *Diploschistes muscorum*) on physiological and biochemical parameters of lichen samples.

Parameter	Independent variables	F	p	η^2	df	Error df	R^2
TBARS	Experimental group	4.64	**0.040**	0.14	1	28	0.86
	Lichen species	167.16	**<0.001**	0.86	1		
	Experimental group × Lichen species	4.66	**0.040**	0.14	1		
EC	Experimental group	8.21	**0.008**	0.23	1	28	0.62
	Lichen species	22.32	**<0.001**	0.44	1		
	Experimental group × Lichen species	14.83	**0.001**	0.35	1		
A_{485}	Experimental group	11.46	**0.002**	0.29	1	28	0.66
	Lichen species	22.14	**<0.001**	0.44	1		
	Experimental group × Lichen species	21.43	**0.000**	0.43	1		
H_2O_2	Experimental group	0.36	0.553	0.01	1	28	0.05
	Lichen species	0.85	0.364	0.03	1		
	Experimental group × Lichen species	0.12	0.733	0.00	1		
Chl *a*	Experimental group	11.88	**0.002**	0.30	1	28	0.62
	Lichen species	27.20	**<0.001**	0.49	1		
	Experimental group × Lichen species	7.49	**0.011**	0.21	1		
Chl *b*	Experimental group	6.23	**0.019**	0.18	1	28	0.31
	Lichen species	1.57	0.221	0.05	1		
	Experimental group × Lichen species	4.51	**0.043**	0.14	1		
Carotenoids	Experimental group	4.91	**0.035**	0.15	1	28	0.76
	Lichen species	80.81	**<0.001**	0.74	1		
	Experimental group × Lichen species	1.99	0.169	0.07	1		
A_{435}/A_{415} ratio	Experimental group	2.58	0.119	0.08	1	28	0.86
	Lichen species	171.63	**<0.001**	0.86	1		
	Experimental group × Lichen species	1.40	0.246	0.05	1		
Rybitol	Experimental group	19.93	**<0.001**	0.42	1	28	0.94
	Lichen species	407.94	**<0.001**	0.94	1		
	Experimental group × Lichen species	0.00	0.978	0.00	1		
Arabitol	Experimental group	3.89	0.059	0.12	1	28	0.94
	Lichen species	459.68	**<0.001**	0.94	1		

	Experimental group × Lichen species	3.75	0.063	0.12	1		
Mannitol	Experimental group	9.11	**0.005**	0.25	1	28	0.85
	Lichen species	146.27	**<0.001**	0.84	1		
	Experimental group × Lichen species	5.20	**0.030**	0.16	1		
GSH	Experimental group	7.24	**0.013**	0.23	1	24	0.78
	Lichen species	78.82	**<0.001**	0.77	1		
	Experimental group × Lichen species	0.02	0.903	0.00	1		
GSSG	Experimental group	6.51	**0.018**	0.21	1	24	0.38
	Lichen species	4.30	**0.049**	0.15	1		
	Experimental group × Lichen species	5.73	**0.025**	0.19	1		
Total glutathione	Experimental group	11.26	**0.003**	0.32	1	24	0.62
	Lichen species	24.34	**<0.001**	0.50	1		
	Experimental group × Lichen species	5.16	**0.032**	0.18	1		

Table S2. The results of two-way analysis of variance ($p < 0.05$) for the effect of the experimental group (control, experimental) and time from hydration (15 min, 3.5 h, 24 h) on the parameters related to photosynthetic efficiency in lichen samples.

**Parameter**	**Species**	**Independent variables**	**F**	**p**	η^2	**df**	**Error df**	R^2
QY_max	*C. aculeata*	Experimental group	10.67	**0.002**	0.20	1	42	0.39
		Time	8.10	**0.001**	0.28	2		
		Experimental group × Time	0.16	0.856	0.01	2		
	D. muscorum	Experimental group	67.33	**<0.001**	0.58	1	49	0.79
		Time	61.26	**<0.001**	0.71	2		
		Experimental group × Time	0.52	0.600	0.02	2		
QY	*C. aculeata*	Experimental group	13.20	**0.001**	0.24	1	42	0.33
		Time	1.09	0.345	0.05	2		
		Experimental group × Time	1.26	0.293	0.06	2		
	D. muscorum	Experimental group	0.001	0.976	0.00	1	49	0.12
		Time	2.99	0.059	0.11	2		
		Experimental group × Time	0.54	0.585	0.02	2		
NPQ	*C. aculeata*	Experimental group	16.10	**<0.001**	0.28	1	43	0.53
		Time	15.02	**<0.001**	0.42	2		
		Experimental group × Time	0.08	0.922	<0.001	2		
	D. muscorum	Experimental group	1.07	0.307	0.02	1	49	0.61
		Time	19.94	**<0.001**	0.45	2		
		Experimental group × Time	15.12	**<0.001**	0.38	2		

QY_max – maximum PSII quantum yield in a dark-adapted sample;
QY – the effective PSII quantum measured in light;
NPQ – the non-photochemical fluorescence quenching in light state.

Table S3. Conditions used in the simulation experiment compared to surface conditions on Mars.

Parameter	Experimental conditions	Mars conditions
Temperature	-26°C – 18°C	Average −55 °C. min. −130 °C. max. 27 °C
Humidity	8-32%	0-100% (Gómez-Elvira et al. 2014)
Pressure	500−700 Pa	677.70−848.25 Pa (Jiang et al. 2023)
Atmospheric gas composition	95% CO_2<br>3.9% N_2<br>1.05% O_2<br>0.05% Ar	95.1% CO_2<br>2.59% N_2<br>1.94 % Ar<br>0.161% O_2<br>0.058% CO<br>(Trainer et al. 2019)

References:

- Gómez-Elvira J, Armiens C, Carrasco I, Genzer M, Gómez F, Haberle R et al. (2014) Curiosity's rover environmental monitoring station: Overview of the first 100 sols. Journal of Geophysical Research: Planets 119, 1680–1688. https://doi.org/10.1002/2013JE004576
- Jiang C, Jiang Y, Li H, Du S (2023) Initial results of the meteorological data from the first 325 sols of the Tianwen-1 mission. Scientific Reports 13, 3325. https://doi.org/10.1038/s41598-023-30513-2
- Trainer M, Wong MH, McConnochie TH et al. (2019) Seasonal variations in atmospheric composition as measured in Gale Crater, Mars. Journal of Geophysical Research: Planets 124, 3000–3024.
